# Genome organization and DNA accessibility control antigenic variation in trypanosomes

**DOI:** 10.1038/s41586-018-0619-8

**Published:** 2018-10-17

**Authors:** Laura S. M. Müller, Raúl O. Cosentino, Konrad U. Förstner, Julien Guizetti, Carolin Wedel, Noam Kaplan, Christian J. Janzen, Panagiota Arampatzi, Jörg Vogel, Sascha Steinbiss, Thomas D. Otto, Antoine-Emmanuel Saliba, Robert P. Sebra, T. Nicolai Siegel

**Affiliations:** 1 Department of Veterinary Sciences, Experimental Parasitology, Ludwig-Maximilians-Universität München, Munich, Germany; 2 Biomedical Center Munich, Department of Physiological Chemistry, Ludwig-Maximilians-Universität München, Planegg-Martinsried, Germany; 3 Research Center for Infectious Diseases, University of Würzburg, Würzburg, Germany; 4 ZB MED – Information Centre for Life Sciences, Cologne, Germany; 5 TH Köln, Faculty of Information Science and Communication Studies, Cologne, Germany; 6 Core Unit Systems Medicine, Institute of Molecular Infection Biology, University of Würzburg, Würzburg, Germany; 7 Department of Physiology, Biophysics & Systems Biology, Rappaport Faculty of Medicine, Technion Israel Institute of Technology, Haifa, Israel; 8 Department of Cell & Developmental Biology, Biocenter, University of Würzburg, Würzburg, Germany; 9 Helmholtz Institute for RNA-based Infection Research, Würzburg, Germany; 10 RNA Biology Group, Institute of Molecular Infection Biology, University of Würzburg, Würzburg, Germany; 11 Wellcome Trust Sanger Institute, Hinxton, Cambridge UK; 12 Centre of Immunobiology, Institute of Infection, Immunity & Inflammation, College of Medical, Veterinary and Life Sciences, University of Glasgow, Glasgow, UK; 13 Icahn Institute and Department of Genetics and Genomic Sciences, Icahn School of Medicine at Mount Sinai, New York, NY USA; 14 Present Address: Centre for Infectious Diseases, Parasitology, Heidelberg University Hospital, Heidelberg, Germany

**Keywords:** Histone Variants, Genome Architecture, Single Molecule Real Time (SMRT), Brucei Genome, Distance-dependent Decay, Parasite genomics, Nuclear organization, Immune evasion, Next-generation sequencing, Histone variants

## Abstract

Many evolutionarily distant pathogenic organisms have evolved similar survival strategies to evade the immune responses of their hosts. These include antigenic variation, through which an infecting organism prevents clearance by periodically altering the identity of proteins that are visible to the immune system of the host^1^. Antigenic variation requires large reservoirs of immunologically diverse antigen genes, which are often generated through homologous recombination, as well as mechanisms to ensure the expression of one or very few antigens at any given time. Both homologous recombination and gene expression are affected by three-dimensional genome architecture and local DNA accessibility^2,3^. Factors that link three-dimensional genome architecture, local chromatin conformation and antigenic variation have, to our knowledge, not yet been identified in any organism. One of the major obstacles to studying the role of genome architecture in antigenic variation has been the highly repetitive nature and heterozygosity of antigen-gene arrays, which has precluded complete genome assembly in many pathogens. Here we report the de novo haplotype-specific assembly and scaffolding of the long antigen-gene arrays of the model protozoan parasite *Trypanosoma brucei*, using long-read sequencing technology and conserved features of chromosome folding^4^. Genome-wide chromosome conformation capture (Hi-C) reveals a distinct partitioning of the genome, with antigen-encoding subtelomeric regions that are folded into distinct, highly compact compartments. In addition, we performed a range of analyses—Hi-C, fluorescence in situ hybridization, assays for transposase-accessible chromatin using sequencing and single-cell RNA sequencing—that showed that deletion of the histone variants H3.V and H4.V increases antigen-gene clustering, DNA accessibility across sites of antigen expression and switching of the expressed antigen isoform, via homologous recombination. Our analyses identify histone variants as a molecular link between global genome architecture, local chromatin conformation and antigenic variation.

## Abstract (web summary)

Long-read sequencing allows the assembly of antigen-gene arrays in *Trypanosoma brucei* and, coupled with deletion experiments, demonstrates that histone variants act as a molecular link between genome architecture, chromatin conformation and antigen variation.

## Main

Genome sequences of several pathogens have revealed a partitioning of chromosomes, with housekeeping genes often being located in the central core and antigen genes being located in subtelomeric regions^5,6^. These assemblies suggest that the linear organization of the genome may be important for restricting high levels of recombination to regions that code for antigens and for ensuring that all but one antigen is repressed.

Recently, genome-wide Hi-C analyses have begun to uncover the 3D organization of chromosomes at high resolution^4^, which has highlighted the critical role of spatial organization and compartmentalization of DNA in the regulation of gene expression and recombination^2,3^. In addition, microscopy-based analyses of the unicellular eukaryotic parasites *Plasmodium falciparum* and *T. brucei* have indicated that nuclear organization may be important for the mutually exclusive expression of antigens^7–9^. However, to our knowledge, the proteins that are involved in shaping genome architecture and controlling antigen expression have not yet been identified in any organism.

This study aimed to identify the process that restricts antigen expression. Specifically, we sought to identify proteins that are important for maintaining genome architecture and to determine whether global and/or local changes in chromatin conformation affect antigen expression.

In *T. brucei*—which is the causative agent of human sleeping sickness—the key antigens are the variant surface glycoproteins (VSGs). Most VSG genes—of which there are about 2,500—are found in long subtelomeric arrays of megabase chromosomes^6^. In addition, about 65 VSG genes are located on mini-chromosomes (50–150 kb in length)^10^ and a smaller subset of VSG genes is located in distinct telomere-proximal polycistronic transcription units, called expression sites^11^. Expression sites are grouped into metacyclic-form and bloodstream-form expression sites (MESs and BESs, respectively) on the basis of the life-cycle stage during which they can be activated. VSG genes are transcribed only when they are located within an expression site and only one of about 15 BESs is transcribed at a time, which ensures that the expression of VSG genes is mutually exclusive^11^. Therefore, a genome sequence that contains both subtelomeric VSG gene arrays and telomeric expression sites, which is lacking in the available *T. brucei* genome (isolate TREU 927)^6^, is required to elucidate the molecular link between genome architecture and antigenic variation.

**Fig. 1:**
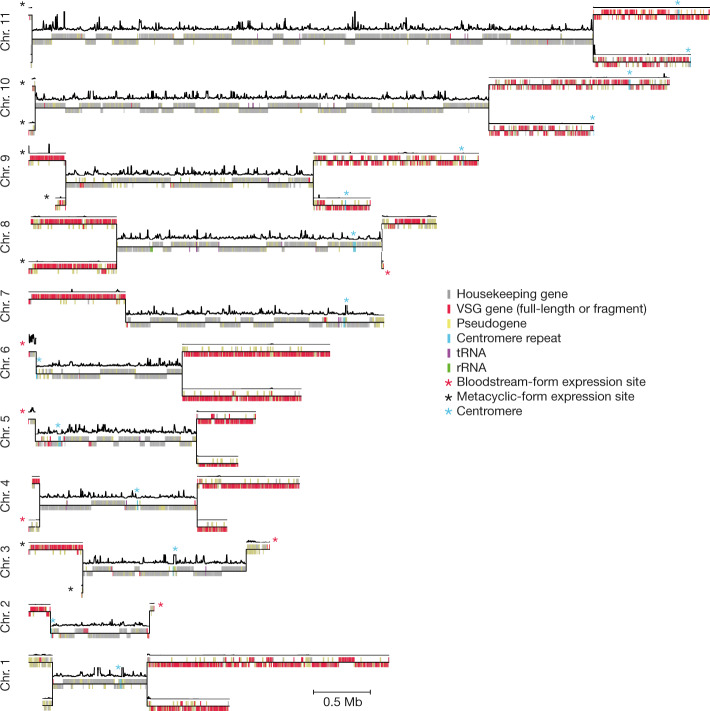
Long-read and Hi-C-based de novo assembly of the *T. brucei* Lister 427 genome. Only one of the two homologous chromosomes (chr.) is depicted for the homozygous chromosomal core regions (22.71 Mb). Both chromosomes are shown for the heterozygous subtelomeric regions (19.54 Mb). Relative transcript levels (window size, 5,001 bp; step size, 101 bp) are shown as a black line above each chromosome. BESs and MESs were assigned to the respective subtelomeric region if an unambiguous assignment based on DNA interaction data was possible (see Supplementary Information). Centromeres were assigned based on KKT2 ChIP–seq data^30^.

**Extended Data Fig. 1:**
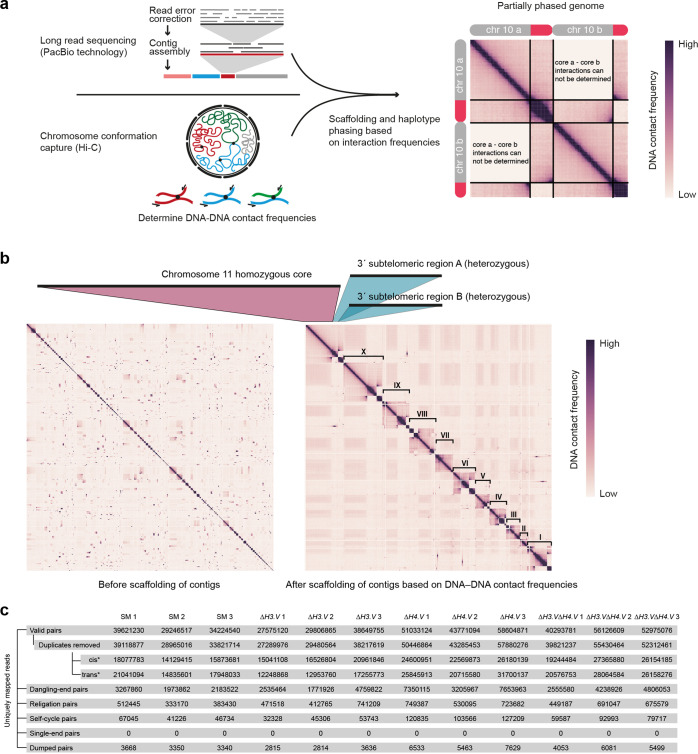
Assembly of the *T. brucei* Lister 427 genome. **a**, Outline of the genome-assembly strategy: gDNA of *T. brucei* Lister 427 was sequenced using SMRT sequencing technology and P6-C4 sequence chemistry. The 10% longest reads were error-corrected using the remaining SMRT reads and assembled into contigs using the HGAPv3 algorithm^41^. Information on spatial contacts between contigs, obtained from Hi-C analyses, was used to position and orient the contigs into scaffolds. **b**, To scaffold and orient the contigs, Hi-C reads were mapped to 1,232 contigs to generate a heat map of DNA–DNA interactions (left). Scaffolding was performed by placing contigs such that the interaction signal located away from the diagonal could not be further reduced (right). Heterozygous subtelomeric regions displayed strong interactions with the chromosomal core region but not with other subtelomeric regions, which indicates that they belong to independent homologous chromosomes. Note that for the left arm of chromosome 7, the heterozygous subtelomeric regions of the two homologous chromosomes could not be assembled separately. **c**, Statistics of Hi-C data analysis based on reads mapped to a joined genome version (haploid A-forks joined to the core). This implies an underestimation of *cis*, and overestimation of *trans* interactions (marked with asterisks), as the B-forks remain un-joined.

**Extended Data Fig. 2:**
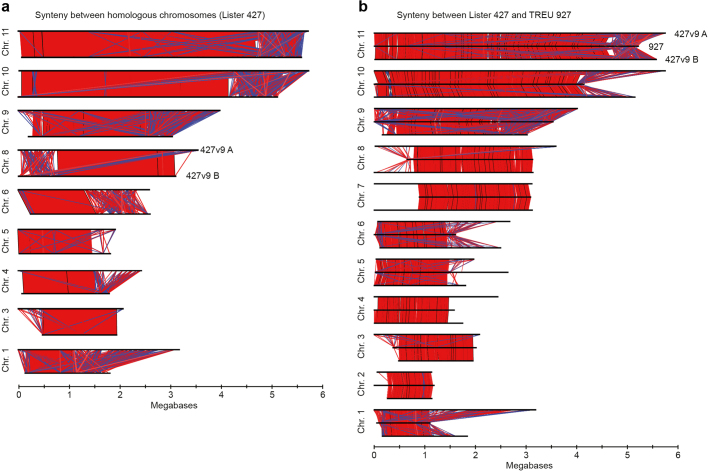
Synteny between homologous chromosomes and between different isolates. **a**, Pairwise comparison of corresponding homologous chromosomes using the Artemis Comparison Tool (ACT) of the Wellcome Trust Sanger Institute^60^. Pairs of regions that share a high degree of similarity (BLAST score ≥ 5,000) are connected by boxes in red, or in blue if they are inverted. Chromosome 7 is not shown because the subtelomeric regions of the two homologous chromosomes are very similar and could not be resolved during the assembly. Chromosome 2 is not shown as only one of the two homologous chromosomes contains an extended subtelomeric region. **b**, Pairwise comparison of the eleven megabase-chromosomes of the TREU 927 isolate (middle black bar) and the corresponding two homologous chromosomes of the Lister 427 isolate (top and bottom black bars) using ACT^60^. Regions that reached a BLAST score of at least 5,000 are drawn in red, or in blue if they are inverted.

Using PacBio single-molecule real-time (SMRT) sequencing technology, we generated an approximately 100-fold genome-sequence coverage of the *T. brucei* 427 Lister isolate (the most commonly used laboratory isolate) and assembled the reads into megabase chromosomes, of which there are 11 (96 contigs, Fig. 1, Extended Data Table 1). To order and orient contigs without relying on scaffolds of related parasite isolates (which may have undergone genome rearrangements), we took advantage of two ubiquitous features of chromosome organization: a distance-dependent decay of DNA–DNA interaction frequency and substantially higher interaction frequencies between DNA loci located on the same chromosome, compared to those on different chromosomes^4^. The high degree of subtelomeric heterozygosity enabled us to assemble the complete *T. brucei* genome with phased diploid subtelomeric regions (Extended Data Figs. 1, 2, Supplementary Data). In addition, RNA sequencing (RNA-seq) revealed a notable partitioning of the genome into a transcribed homozygous core and non-transcribed heterozygous subtelomeric regions, which encode the vast repertoire of antigens (Fig. 1).

**Fig. 2:**
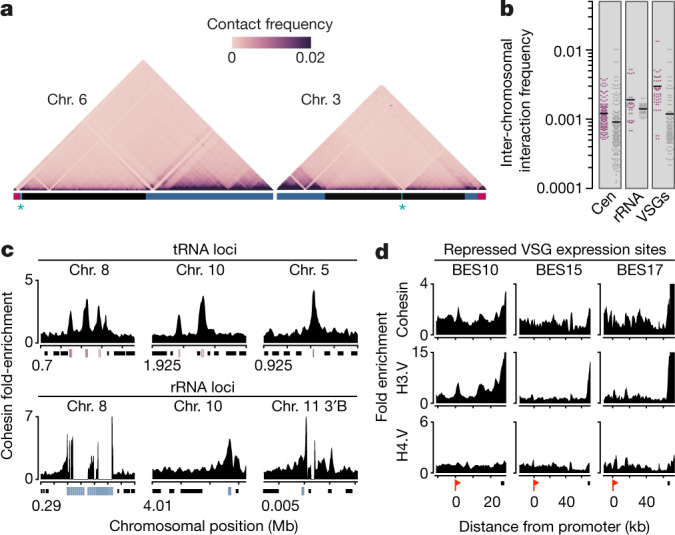
Hi-C and ChIP–seq reveal partitioning of the *T. brucei* genome into distinct domains. **a**, Hi-C heat maps of chromosomes 3 and 6 at 20-kb resolution. Horizontal blue, black and red lines mark heterozygous subtelomeric, homozygous core regions and BESs, respectively. Centromeres are marked by asterisks. **b**, Scatter plot showing inter-chromosomal interaction frequencies among centromeres (cen) (*n* = 206 bins; *P* = 0.0029), VSG genes in silent expression sites (VSGs) (*n* = 54 bins; *P* = 1.63 × 10^−6^) and rRNA genes (*n* = 40 bins; *P* = 0.0177) compared to a matching background sample, which was randomly selected from the interaction matrix (50-kb bin size). The background sample (grey) matches the genomic feature (red) in size and number. Selected bins with zero values were removed from both the query and background sample. *P* values are based on Welch’s *t*-test (two-sided). Black lines represent the mean. **c**, ChIP–seq data showing the enrichment (compared to input material) of the cohesin subunit SCC1 (*n* = 3 biologically independent experiments) across representative tRNA and rRNA genes (window size, 501 bp; step size, 101 bp). Black, red and blue boxes represent protein coding, tRNA and rRNA genes, respectively. Tick marks on the *x* axis represent 5-kb intervals. 3′B refers to one of the two alternative subtelomeric ends (A or B) at the 3′end of chromosome 11. **d**, ChIP–seq data showing cohesin (*n* = 3 biologically independent experiments), H3.V and H4.V (each in *n* = 2 biologically independent experiments) enrichment across three transcriptionally repressed BESs (window size, 2,001 bp; step size, 501 bp). Red flags mark BES promoters and black boxes indicate the locations of VSG genes.

**Extended Data Fig. 3:**
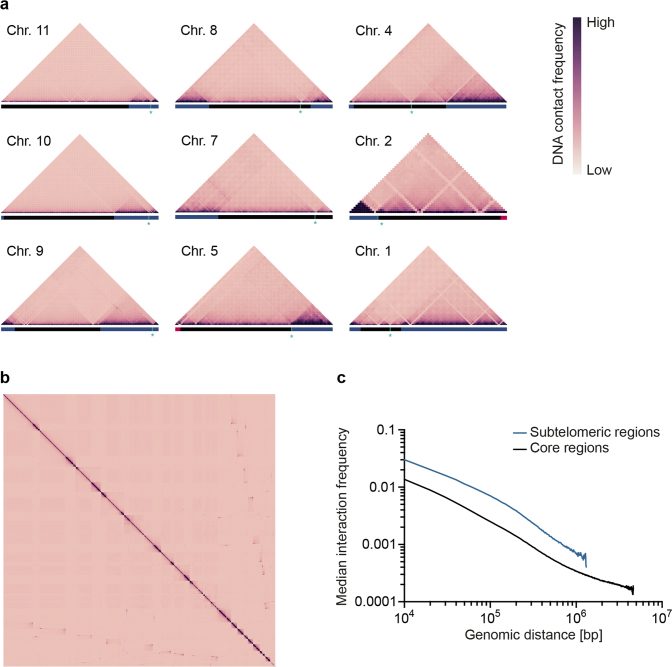
Compartmentalization of megabase chromosomes in wild-type cells. **a**, Hi-C heat maps of individual chromosomes at 20-kb resolution. Horizontal lines mark subtelomeric regions (blue), core regions (black) and bloodstream-form expression sites (red). A blue vertical line and an asterisk indicate the locations of centromeres. **b**, Hi-C heat map of the haploid genome with one set of subtelomeric regions joined to the core regions (20-kb resolution). **c**, Decay of frequency of intra-chromosomal contacts as a function of genomic distance (20-kb bin size) within subtelomeric (blue) and core (black) regions. The median across the core (*n* = 11) and subtelomeres (*n* = 32) is shown.

Analysis of the frequency of intra-chromosomal DNA–DNA interaction suggested a strong compartmentalization of the *T. brucei* genome: centromeres and junctions between the core and subtelomeres function as the most prominent boundaries of DNA compartments. In addition, the frequency of DNA–DNA contact was substantially higher across subtelomeric regions compared to core regions, which indicates that subtelomeres are more compact than the core region (Fig. 2a, Extended Data Fig. 3). Therefore, the partitioning of the genome into transcribed housekeeping genes and non-transcribed antigen genes that is observed in the genome assembly and transcriptome data is mirrored by the 3D organization of the genome. In *T. brucei*, RNA polymerase II transcription can occur in the absence of canonical promoter motifs^12,13^. Thus, the high degree of compaction across subtelomeric regions probably prevents the spurious initiation of transcription and ensures mutually exclusive expression of a single VSG gene from a BES. In addition, BES–BES interactions were much more frequent than interactions among randomly chosen genomic loci, suggesting a clustering of BESs (Fig. 2b). Taken together, the Hi-C data suggest a distinct compartmentalization of the *T. brucei* nucleus.

Higher-order genome structures are established and maintained by architectural proteins such as CCCTC-binding factor (CTCF) and cohesin^14^. Histone variants are also enriched at many compartment boundaries^15^, but the role of these variants in shaping genome architecture remains unknown. Although CTCF appears to be absent in non-metazoans^16^, the major subunit of cohesin (SCC1) is present in *T. brucei* and the depletion of this subunit causes deregulation of VSG expression^17^. However, it has remained unclear whether this is a direct effect because SCC1 depletion strongly affects cell-cycle progression and growth rate, leading to rapid parasite death^18^.

**Extended Data Fig. 4:**
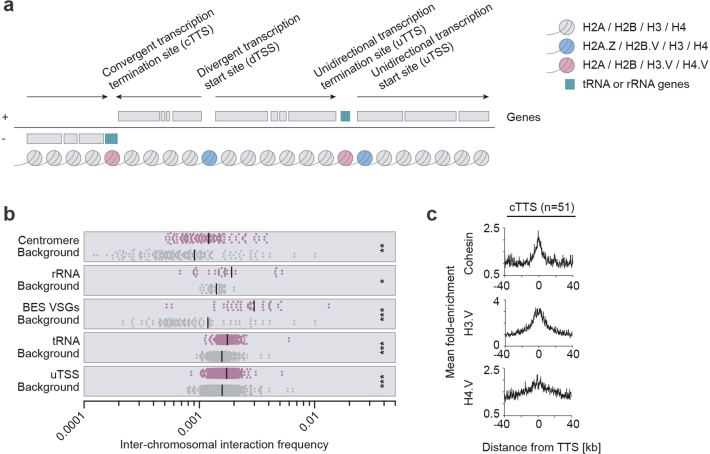
Hi-C and ChIP–seq reveal partitioning of the *T. brucei* genome into distinct domains. **a**, Outline of the genome organization. Boundaries of transcription units are marked by nucleosomes that contain different types of histone variants. Black arrows indicate the direction of transcription. **b**, Scatter plot showing inter-chromosomal interactions among centromeres (*n* = 206 query bins, *n* = 292 background bins, *P* = 0.0029), VSG genes (*n* = 54 query bins, *n* = 130 background bins, *P* = 1.63 × 10^−6^), rRNA genes (*n* = 40 query bins, *n* = 64 background bins, *P* = 0.0177), tRNA genes (*n* = 614 query bins, *n* = 620 background bins, *P* = 2.45 × 10^−190^) and unidirectional transcription start sites (*n* = 3,142 query bins, *n* = 3,682 background bins, *P* = 6.49 × 10^−91^) compared to a background sample, which was randomly selected from the interaction matrix (50-kb bin size). The background sample matches the genomic feature in size and number. Selected bins with zero values were removed from both the query and background sample. *P* values are based on Welch’s *t*-test (two-sided). Black lines represent the mean. **c**, ChIP–seq data showing cohesin, H3.V and H4.V enrichment (compared to input material) averaged across all convergent transcription termination sites (cTTS, *n* = 51) (window size, 101 bp; step size, 11 bp).

Chromatin immunoprecipitation with sequencing (ChIP–seq) revealed that in *T. brucei* SCC1 is enriched across tRNA and rRNA genes, termination sites of RNA polymerase II transcription and most of the 3′ ends of BESs (Fig. 2c, d, Extended Data Fig. 4). This pattern of cohesin enrichment is reminiscent of its distribution in humans and yeast, in which cohesin is found at insulator and boundary elements such as tRNA genes^19,20^. The observed distribution of SCC1 is also similar to that of histone variants H3.V and, to a lesser extent, H4.V in *T. brucei* (Fig. 2d, Extended Data Fig. 4; also see ref. ^21^). This raised the possibility that these two histone variants function together with SCC1 in shaping genome organization and the regulation of antigen expression.

**Extended Data Fig. 5:**
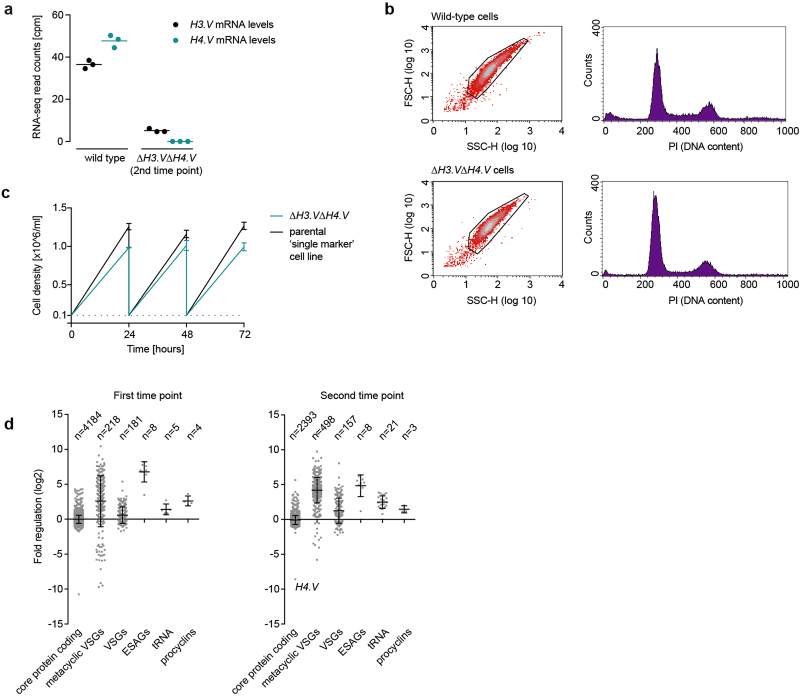
Characterization of Δ*H3.V*Δ*H4.V* cells. **a**, RNA-seq fragment counts on *H3.V* and *H4.V* CDS in wild-type and Δ*H3.V*Δ*H4.V* cells, normalized by million fragments mapped to protein-coding genes. Note that the first and last codon of the *H3.V* open reading frame were not deleted. As a result, a small number of *H3.V* reads are detected even in the Δ*H3.V* cells. **b**, Cell-cycle analysis based on flow cytometry, of wild-type and Δ*H3.V*Δ*H4.V* cells. One of three replicates is shown. **c**, Growth curve (mean ± s.d.) of wild-type and Δ*H3.V*Δ*H4.V* cells (*n* = 3 biologically independent replicates). **d**, RNA-seq of Δ*H3.V*Δ*H4.V* cells (first and second time points). The mean ± s.d. fold change in expression compared to wild type (*n* = 3 biologically independent experiments) is shown, for the significantly regulated genes (based on a Benjamini–Hochberg adjusted *P* value from a two-sided Wald test with false discovery rate < 0.1) from different gene groups. ESAGs, expression-site-associated genes.

To investigate a possible link between these histone variants, genome architecture and antigen expression, we determined the expression of VSG genes and genome architecture in Δ*H3.V*, Δ*H4.V* and Δ*H3.V*Δ*H4.V* cells. No cell cycle defect was observed in these cell lines (Extended Data Fig. 5).

**Fig. 3:**
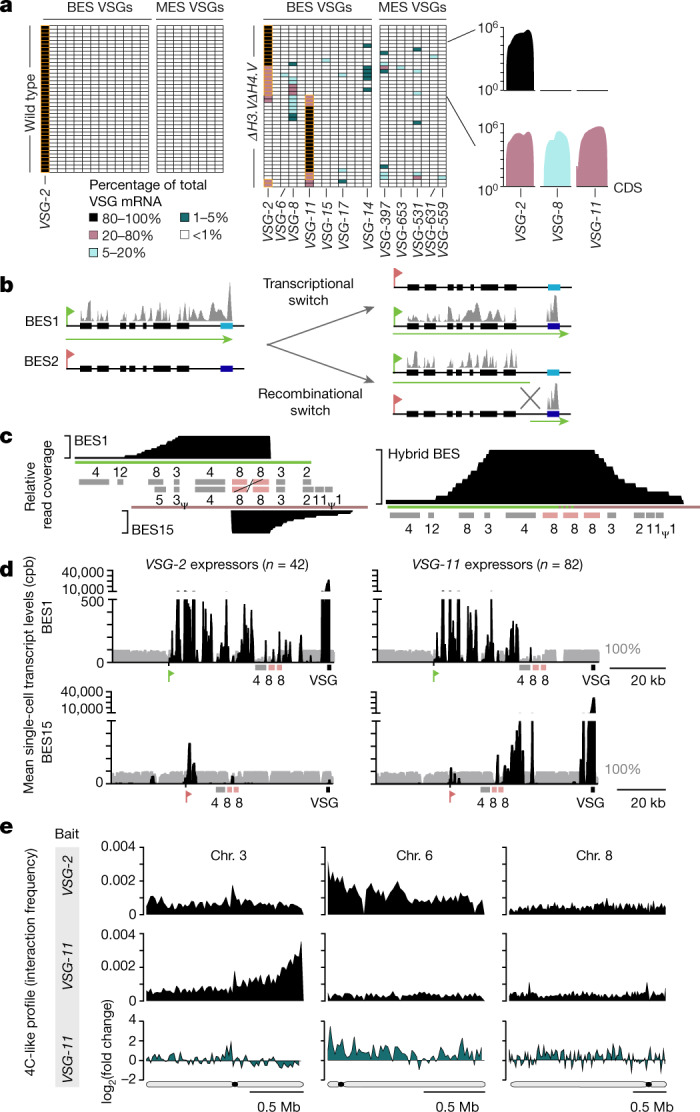
Deletion of histone variants *H3.V* and *H4.V* leads to a switch in expression of VSG isoforms. **a**, scRNA-seq analysis of wild-type (*n* = 40) and Δ*H3.V*Δ*H4.V* (first time point, *n* = 44) cells. Each row represents data from one cell. The number of sequencing reads was normalized to account for differences in library size, gene length and uniqueness of VSG gene sequence. Only uniquely mapping reads were considered. The total number of VSG transcripts per cell is set to 100% (for details, see Methods, Extended Data Fig. 7). The colour code indicates the contribution of individual VSG transcripts to the pool of VSG transcripts in a single cell. The dominant VSG isoform is depicted with an orange border. For selected cells, the read coverage is shown across *VSG-2*, *VSG-8* and *VSG-11* (with 500 bp of surrounding sequence). CDS, coding sequence. **b**, Outline of the VSG switching mechanisms described for *T. brucei*. Green and red flags mark the active and repressed promoters, respectively. Green lines and grey bars indicate regions of expected transcription for the two different scenarios. **c**, Sequencing coverage across BES1 (left, top), BES15 (left, bottom) and a hybrid BES consisting of the 5′-BES1 and 3′-BES15 (right). Coverage is based on SMRT sequencing reads >10 kb from Δ*H3.V*Δ*H4.V* gDNA that map to BES1 and BES15. The cross represents the site of recombination. Boxes represent expression-site-associated genes and ψ denotes a pseudogene. **d**, scRNA-seq-based analysis of Δ*H3.V*Δ*H4.V* cells that exclusively express *VSG-2* (*n* = 42) or *VSG-11* (*n* = 82). The average transcript levels (counts per billion, cpb) based on uniquely mapping reads across BES1 and BES15 are shown. Grey bars represent degree of uniqueness. **e**, 4C-like inter-chromosomal interaction profiles (based on Hi-C-data, 20-kb bin size) showing the average interaction frequencies of BES1 (top) and BES15 (middle) with chromosomes 3, 6 and 8 in wild-type cells and the fold change (log_2_) in interactions of BES15 (bottom) with chromosomes 3, 6 and 8 after deletion of *H3.V* and *H4.V*.

Laboratory-adapted isolates, such as the one used here, switch their expression of VSG isoforms at very low frequency (about 10^−6^ per population doubling), and homogenously express *VSG-2* (Fig. 3a; also see ref. ^22^). Thus, an increase in heterogeneity of VSG gene expression can be caused by a loss of mutually exclusive expression of VSG genes in individual cells—that is, heterogeneity in antigen expression at the single-cell level—or an increased switching frequency in expression of VSG genes in different parasites (heterogeneity at the population level).

**Extended Data Fig. 6:**
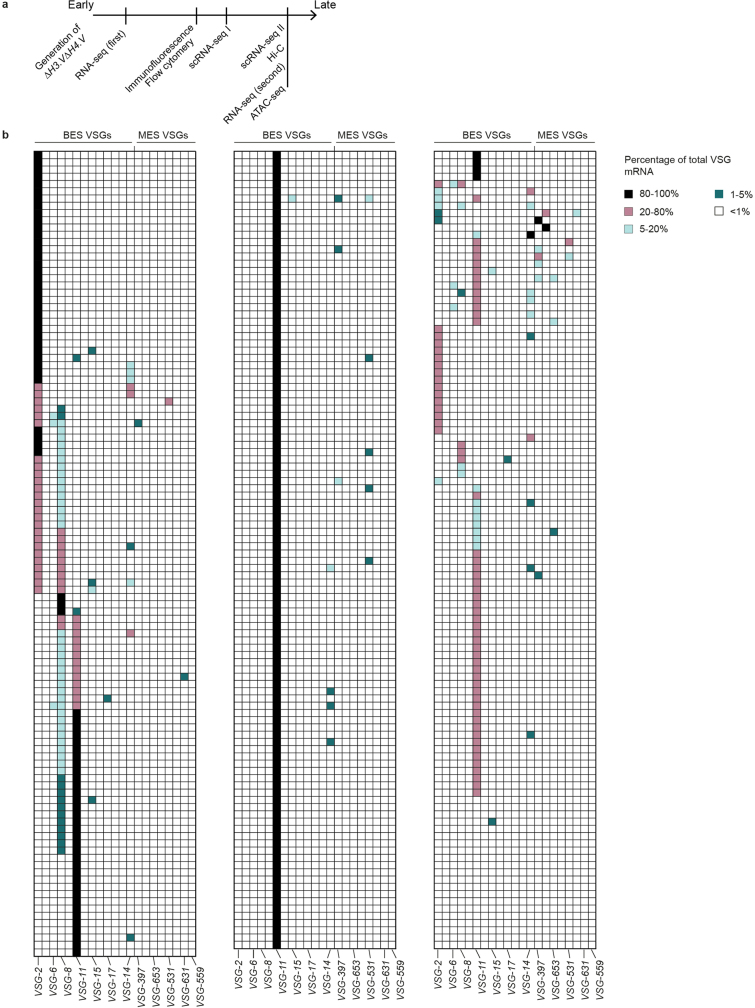
Analysis of Δ***H3.V***Δ***H4.V*** cells. **a**, Order of cell analyses. **b**, scRNA-seq analyis of Δ*H3.V*Δ*H4.V* cells, at the second time point. (*n* = 338). Each row represents data from one cell. For details, see Fig. 3a, Extended Data Fig. 7.

**Extended Data Fig. 7:**
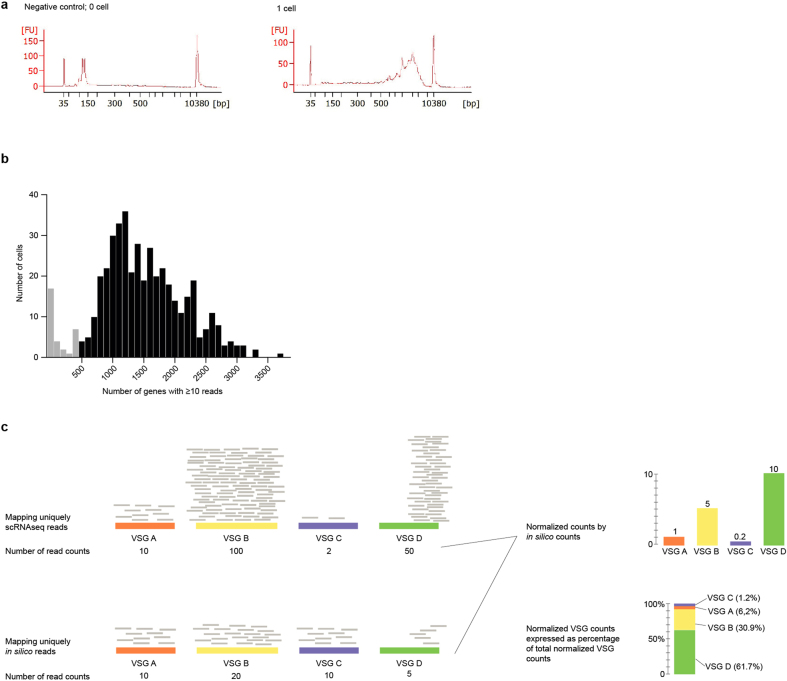
scRNA-seq quality control, VSG gene normalization and quantification. **a**, Representative Bioanalyzer profiles (Agilent) of cDNA from 0 cells (*n* = 6) and 1 cell (*n* = 18, supplemented with ERCC spike-in control). **b**, Histogram representing the total number of genes expressed per single cell (wild type and Δ*H3.V*Δ*H4.V*; *n* = 452). Cells with fewer than 500 genes (grey bars) were excluded from the analysis. **c**, Diagram representing quantification of expression of VSG genes, and the normalization procedure. The reads obtained in each single-cell library were mapped to the genome, keeping only the uniquely mapping reads (mapq > 0). Next, the number of reads mapping to each VSG gene was quantified. To account for differences in length and ‘uniqueness’ among the different VSG genes, the same procedure was performed with an in silico set of reads. The read counts to each VSG gene in each scRNA-seq assay were normalized for ‘uniqueness’ and gene length by dividing them by the counts obtained with the in silico dataset. Finally, for each cell the normalized read counts for each VSG gene were expressed as a percentage of the total number of normalized counts to VSG genes.

**Extended Data Fig. 8:**
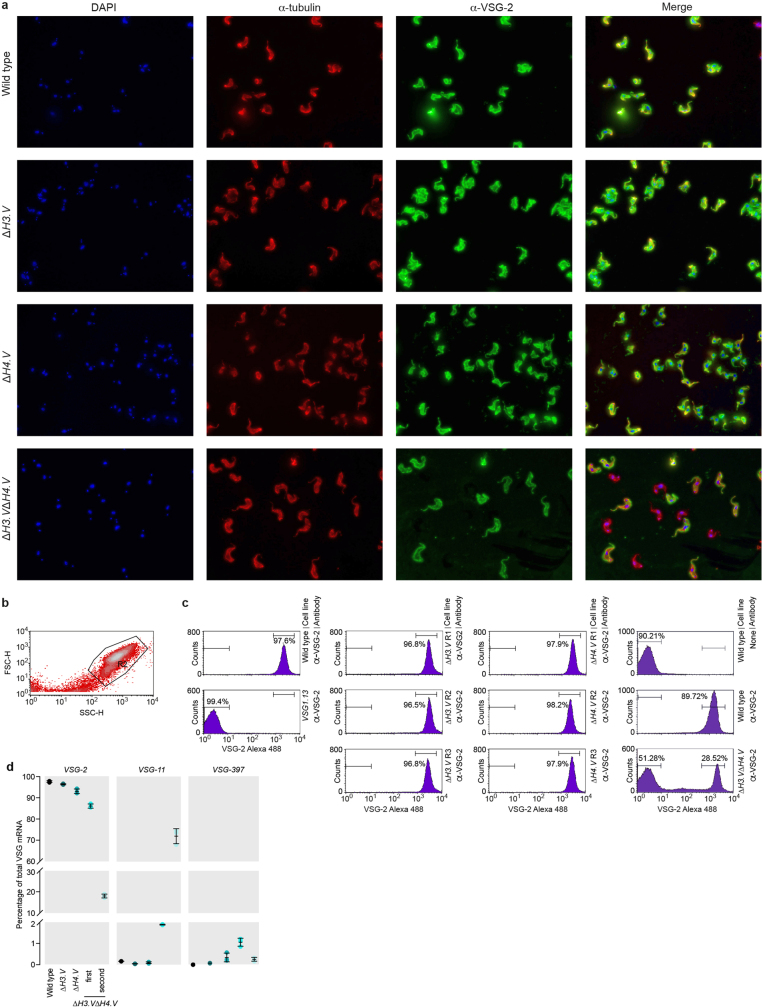
Mutually exclusive expression of VSG genes is not lost in Δ*H3.V* and Δ*H4.V* single-knockout cells. **a**, Immunofluorescence imaging in wild-type, Δ*H3.V*, Δ*H4.V* and Δ*H3.V*Δ*H4.V* cells (*n* = 1). Representative images of 26–28 stacks (0.1976-μm voxel size, maximum projection) are shown. Scale bar, 10 μm. **b**, Gating strategy used for all analyses. **c**, Flow cytometry analysis of *VSG-2* expression in Δ*H3.V*, Δ*H4.V* and Δ*H3.V*Δ*H4.V* cells. Wild-type cells were used as a *VSG-2* positive control, and cells expressing *VSG-13* were used as a negative control. Δ*H3.V*, *n* = 3; Δ*H4.V*, *n* = 3; Δ*H3.V*Δ*H4.V*, *n* = 7 (measured at different time points). For each assay, 50,000 events were gated. **d**, Heterogeneity in expression of VSG genes, based on RNA-seq. The contributions of the dominant *VSG-2* and two additional VSG genes found to be upregulated in Δ*H3.V*Δ*H4.V* cells relative to the total VSG mRNAs are depicted. For each condition, mean mRNA levels and s.d. are derived from *n* = 3 biologically independent RNA-seq experiments.

To distinguish between these possibilities and to identify the VSG genes that are expressed, we performed single-cell RNA-seq (scRNA-seq) of individual *T. brucei* cells. scRNA-seq data from a total of 40 wild-type and 378 Δ*H3.V*Δ*H4.V* cells revealed that—whereas all wild-type cells expressed *VSG-2*—in 74% of the Δ*H3.V*Δ*H4.V* cells, *VSG-2* transcript levels contributed less than 20% of the total VSG mRNA; this indicates a switch in expression of VSG genes (Fig. 3a, Extended Data Figs. 6, 7). Activation of new VSG genes was not random, with *VSG-11* being the dominant newly activated VSG gene in 230 out of 378 cells. In addition, several cells contained transcripts from multiple VSG genes, which points to a partial loss of mutually exclusive expression. To determine the stability of *VSG-2* expression, we analysed Δ*H3.V*Δ*H4.V* cells at two time points that were about 50 population doublings apart. Although the overall pattern remained the same (Fig. 3a, Extended Data Fig. 6), the percentage of cells that expressed only *VSG-2* mRNA, or multiple VSG mRNAs, had declined by the second time point. This suggests that the process of *VSG-2* deactivation had progressed further, and that the simultaneous expression of multiple VSG genes may have been a transient intermediate state. Analyses based on immunofluorescence and flow cytometry confirmed that the loss of *VSG-2* mRNA resulted in a loss of *VSG-2* expression (Extended Data Fig. 8). No major effect on the expression of VSG genes was observed upon deletion of *H3.V* or *H4.V* alone (Extended Data Fig. 8).

In *T. brucei*, the switching of expression of VSG genes occurs by two distinct mechanisms^11^: either by switching transcription from one BES to another (transcriptional switch) or by a recombination-based event that leads to the replacement of the previously active VSG gene with a new VSG gene from a different genomic location (recombinational switch, Fig. 3b).

To gain insight into the mechanism by which histone variants affect antigen expression, we sequenced Δ*H3.V*Δ*H4.V* genomic DNA using SMRT sequencing technology. The SMRT data indicated that, in most cells, recombination had occurred between an expression-site-associated gene 8 (*ESAG8*) gene pair that was present in both BES1 and BES15. The data also revealed that the new chimeric BES contained three copies of *ESAG8*, one from BES1 and two from BES15 (Fig. 3c). scRNA-seq and Hi-C data support a recombination event (Fig. 3d, e). Hi-C data revealed that, upon deletion of *H3.V* and *H4.V*, the interaction frequency between *VSG-11* and the 5′ end of chromosome 6—where *VSG-2* is located in wild-type cells—increased, indicating that *VSG-11* had relocated to chromosome 6.

**Fig. 4:**
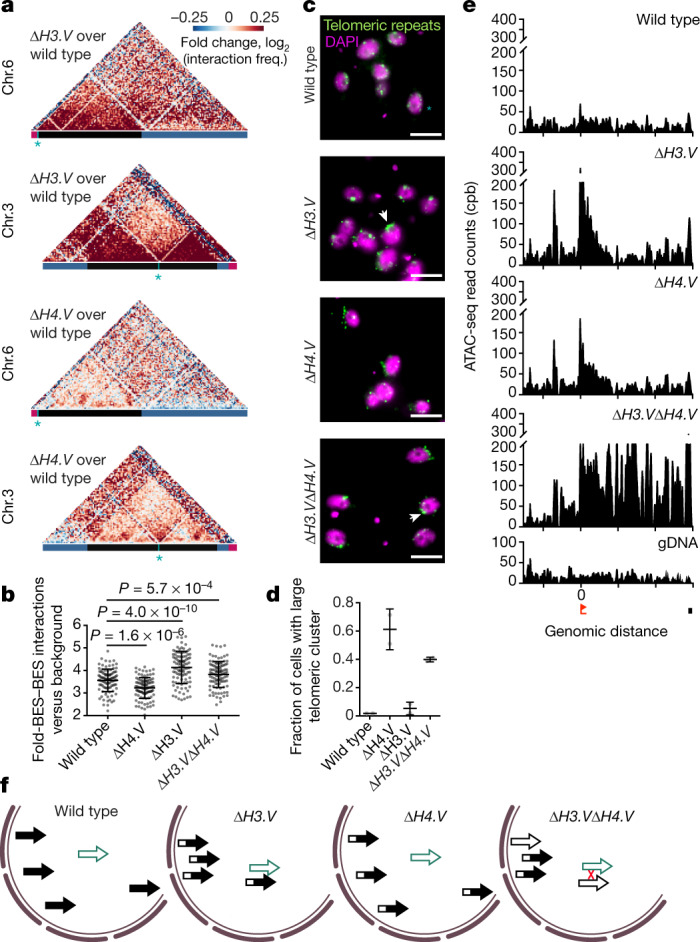
Histone variants H3.V and H4.V influence global and local chromatin structures. **a**, Hi-C heat map showing the fold change (log_2_) in DNA–DNA interaction frequency between wild-type and Δ*H3.V* (top two panels) and Δ*H4.V* cells (bottom two panels). **b**, Fold change in DNA–DNA interaction frequency among VSG genes located in BESs compared to background DNA–DNA interactions in wild-type, in Δ*H4.V*, Δ*H3.V* and Δ*H3.V*Δ*H4.V* cells. The ratios for each cell line were calculated for 100 randomly selected background regions. Mean ± s.d. Significance was determined using Welch’s *t*-test (two-sided). **c**, FISH with probes against telomeric repeats (Alexa Fluor488, green), *n* = 2 biologically independent experiments. Scale bar, 5 μm. **d**, Quantification of telomere signal to determine the fraction of cells containing large telomeric clusters (white arrows in **c**) was performed using Imaris 8 and is based on the analysis of 1,128 cells. Means ± s.d. of two replicates are shown (wild type: *n* = 116, 221; Δ*H3.V*: *n* = 140, 102; Δ*H4.V*: *n* = 146, 107 and Δ*H3.V*Δ*H4.V*: *n* = 190, 106). **e**, ATAC-seq data (*n* = 2 biologically independent experiments) across BES15 (repressed in wild-type cells). gDNA read coverage (bottom) is shown to illustrate mappability of reads (window size, 501 bp; step size, 101 bp). Red flag and black box indicate the position of the promoter and the VSG gene, respectively. Tick marks on *x* axes represent 20-kb intervals. **f**, Model illustrating the influence of H3.V and H4.V on genome architecture and local DNA accessibility. H3.V and H4.V single knockouts alone mediate only partial opening of BESs (half open arrow) and H3.V knockout leads to a spatial rearrangement of BESs inside the nucleus, whereas deletion of both histone variants is required to obtain the fully opened BESs (open arrow) and spatial proximity of BESs that facilitate recombination (red cross) and lead to the expression of a new VSG isoform.

**Extended Data Fig. 9:**
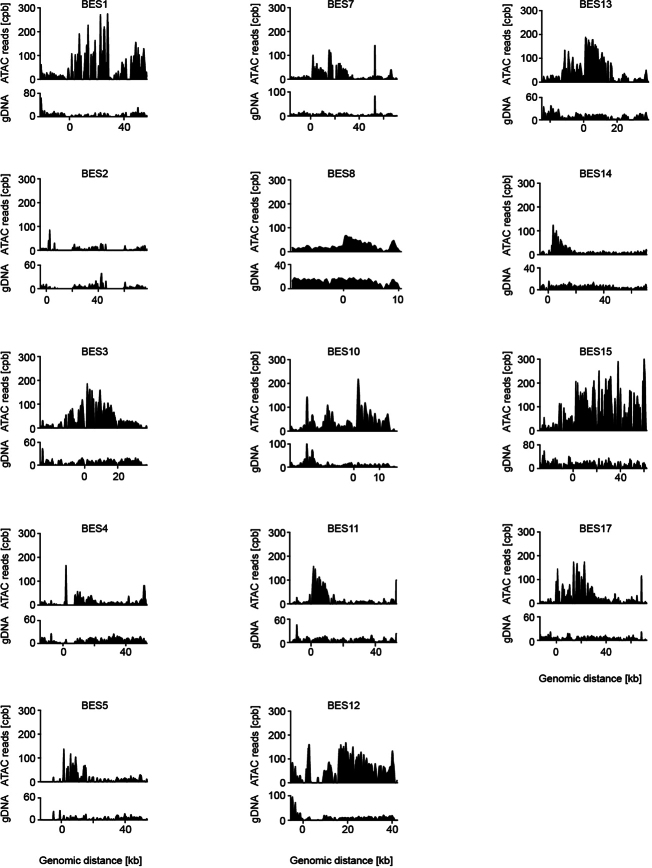
DNA accessibility across BESs in Δ*H3.V*Δ*H4.V* cells. Uniquely mapping ATAC-seq reads across all BESs are shown. The 0-nt position corresponds to the promoter. Uniquely mapping gDNA-seq reads are shown to illustrate differences in mappability. For ATAC-seq, *n* = 2 biologically independent experiments were performed, using sample material from 10 million cells in one experiment and from 20 million cells in the other.

Studies in different organisms have shown that the frequency of recombination is affected by spatial proximity and DNA accessibility^23,24^. Thus, to determine whether histone variants contribute to genome architecture and/or local DNA accessibility, we performed Hi-C and assays for transposase-accessible chromatin using sequencing (ATAC-seq). Hi-C data from Δ*H3.V* cells revealed marked changes in inter-chromosomal interactions (Fig. 4a, top) and a significant increase in interactions among repressed BESs (Fig. 4b), pointing to a loss of constraints that may have ‘anchored’ the BESs to specific nuclear sites. In support of these Hi-C data, fluorescence in situ hybridization (FISH) data revealed a strong clustering of telomeric repeats upon deletion of *H3.V* (Fig. 4c, d). By contrast, deletion of *H4.V* affected genome architecture only modestly (Fig. 4a, bottom). Unlike the Hi-C data, our ATAC-seq data indicated that promoter-proximal DNA accessibility increased upon *H3.V* or *H4.V* deletion (Fig. 4e). However, only Δ*H3.V*Δ*H4.V* cells exhibited high DNA accessibility across the entire length of transcriptionally repressed BESs (Fig. 4e bottom, Extended Data Fig. 9).

In summary, the Hi-C and ATAC-seq data indicate that although deletion of *H3.V* was responsible for the majority of genome architectural changes and increased BES clustering, this alone was not sufficient to induce a switch in expression of VSG genes. Only the concurrent deletion of *H3.V* and *H4.V*, which also strongly increased DNA accessibility across transcriptionally repressed BESs, enhanced the rate of recombination-based switching of VSG genes.

The depletion of histone H3 was previously shown to upregulate BES proximal-promoter activity—presumably via a general increase in DNA accessibility—but did not cause deregulation of VSG genes^25^. We hypothesize that the marked increase in switching frequency of VSG gene expression results from the combination of decreased spatial distance between BESs and increased local DNA accessibility (Fig. 4f).

The activation of new VSG genes did not occur at random; this non-random activation has previously been observed for infections of different hosts^26,27^. In a small number of cells, we detected transcripts from different VSG isoforms. This loss of mutually exclusive expression of VSG genes may be caused by increased DNA accessibility upon the deletion of histone variants, which may result in promiscuous RNA polymerase II transcription. Our observations that even in Δ*H3.V*Δ*H4.V* cells not all expression sites are transcribed and that specific ‘pairs’ of VSG genes tend to be co-expressed, suggest that there are additional constraints imposed by genome organization or VSG protein structure^28^. At the genome level, co-activated VSG genes may have to be localized in close proximity to ensure sufficient levels of an activating factor^8,29^; alternatively, differences in VSG protein structure may make it impossible for the parasite to tolerate certain mosaic surface coats.

In this study, we have demonstrated how evolutionarily conserved features of genome architecture can be exploited for the de novo scaffolding of phased diploid genomes. The use of Hi-C, scRNA-seq and ATAC-seq—to our knowledge, all used here for the first time in *T. brucei*—opened opportunities for genome assembly and the characterization of the mechanism that underlies VSG switching in Δ*H3.V*Δ*H4.V* cells. Our data reveal that histone variants can function as architectural proteins, and that changes in global genome architecture and local chromatin configuration can induce extensive switches in antigen expression.

## Methods

No statistical methods were used to predetermine sample size. The experiments were not randomized and investigators were not blinded to allocation during experiments and outcome assessment.

### Cell culture

All *T. brucei* strains used in this study are derivatives of the Lister 427 bloodstream-form isolate. Cells were cultured in HMI-11 medium (HMI-9^31^ without serum plus) at 37 °C up to a density of 10^6^ cells/ml. If required, drugs were used at standard concentrations.

### Cell lines

Δ*H3.V* and Δ*H4.V* cells used in this study have previously been published^21,32^. After generation of a transgenic cell line, the correct tagging of a gene or the deletion of gene was verified by PCR. Cell lines were not tested for mycoplasma contamination.

Ty1-H3.V/ΔH3.V *cells*. To delete the first *H3.V* allele (Tb927.10.15350), the regions upstream and downstream of the *H3.V* CDS were PCR-amplified using the following primer pairs: H3.V_01_F, H3.V_02_R and H3.V_03_F, H3.V_04_R (see Supplementary Table 4 for a full list of oligonucleotides) and cloned into pyrFEKO-Puro^33^ using InFusion HD Cloning Plus reagents (Clontech) at PvuII/HindIII and BamHI/SbfI restriction sites. The resulting plasmid was linearized with PvuII and SbfI and stably transfected into the *H3.V* locus of *T. brucei* wild-type cells to generate *H3.V*/Δ*H3.V* cells. To add an N-terminal 2× Ty1 tag to the second *H3.V* allele, the sequence of 326-bp upstream of the *H3.V* CDS (H3.V_05_F, H3.V_06_R) was cloned into the ApaI/NotI linearized vector pPOTv3-2×Ty1 using InFusion HD Cloning Plus reagents (Clontech). Downstream of the blasticidin resistance marker and the Ty1-tag, a 417-bp DNA sequence homologous to the *H3.V* CDS 5′-end (H3.V_07_F, H3.V_08_R) was amplified (leaving out the ATG start codon) and likewise inserted using SacI and NheI restriction sites. The tag sequence was subsequently replaced by a codon-optimized version: oligonucleotides containing two codon-optimized Ty1 coding sequences (H3.V_09 and H3.V_10) were annealed, digested with HindIII and SacI and ligated into the HindII/SacI-linearized plasmid. Finally, the plasmid was linearized with ApaI and NheI restriction enzymes and stably transfected into *H3.V*/Δ*H3.V* cells to generate *Ty1-H3.V/*Δ*H3.V*.

ΔH3.VΔH4.V *double-knockout cells*. To delete *H4.V*, the upstream (H4.V_11_F, H4.V_12_R) and downstream (H4.V_13_F, H4.V_14_R) regions flanking the *H4.V* CDS (Tb927.2.2670) were amplified from bloodstream-form wild-type gDNA and purified using NucleoSpin Gel and PCR Clean-up kit (Macherey-Nagel). The PCR product of the upstream region was inserted into the plasmid pyrFEKO-Neo^33^ using InFusion HD Cloning Plus reagents (Clontech) between HindIII and AgeI restriction sites. The downstream region was integrated by ligation using BamHI and SbfI restriction sites. The neomycin resistance cassette was exchanged with a blasticidin or a phleomycin resistance marker, respectively, using the BglII and XbaI sites that flank the resistance marker. To this end, the blasticidin and phleomycin cassettes were excised from pyrFEKO-BSD or pyrFEKO-Phleo, purified and ligated into the target plasmid. The plasmids were linearized with NheI and SbfI and stably transfected into the previously published Δ*H3.V* cell line^32^.

Ty1-SCC1/ΔSCC1 *cells*. To delete the first *SCC1* allele (Tb927.7.6900), the flanking regions upstream (Scc1_15_F, Scc1_16_R) and downstream (Scc1_17_F, Scc1_18_R) of the *SCC1* CDS were amplified, digested with PvuII/HindIII and BamHI/SbfI, respectively, and ligated into pyrFEKO-Hyg^33^ at PvuII/HindIII and BamHI/SbfI restriction sites. Wild-type cells were transfected with the linearized plasmid (PvuII/SbfI) to obtain *SCC1/*Δ*SCC1* cells. For the N-terminal Ty1-tagging of the second *SCC1* allele, the 3′ end of the *SCC1* 5′ UTR was amplified (Scc1_19_F, Scc1_20_R), digested with ApaI and NotI and ligated into the ApaI/NotI-linearized vector pPOTv3-2×Ty1. Next, the 5′ end of the *SCC1* CDS was amplified (leaving out the ATG start codon) (Scc1_21_F, Scc1_22_R), digested with SacI and NheI and ligated into the likewise-digested plasmid. The Ty1-tag was exchanged by a codon-optimized version as described for N-terminal tagging of *H3.V* (see above). The ApaI/NheI linearized plasmid was stably transfected into *SCC1*/Δ*SCC1 T. brucei* cells to generate *Ty1-SCC1/*Δ*SCC1*.

The cell line in which both endogenous *H4.V* alleles are knocked out and ectopic overexpression of a Ty1-tagged version of H4.V can be induced has previously been published^21^.

### In situ Hi-C

Because Δ*H3.V*^32^ and Δ*H4.V*^21^ cells had been generated in a ‘single marker’ background^34^, we generated the Δ*H3.V*Δ*H4.V* cells in a single marker background and compared the Hi-C profiles of the transgenic cell lines to those generated from single marker cells. Thus, all ‘wild-type’ Hi-C data shown in Figs. 2–4 and Extended Data Figs. 3, 4 are generated from single marker cells. Hi-C data from ‘true’ wild-type cells (Lister 427, MiTat 1.2) were also generated, but used only for the genome assembly.

In situ Hi-C was performed based on previously published protocols^35,36^ and adapted to *T. brucei*: 2 × 10^8^ cells (wild type, single marker, Δ*H3.V*, Δ*H4.V* and Δ*H3.V*Δ*H4.V*) were collected and resuspended in 40 ml of 1× trypanosome dilution buffer (1× TDB; 0.005 M KCl, 0.08 M NaCl, 0.001 M MgSO_4_ ×7H_2_O, 0.02 M Na_2_HPO_4_, 0.002 M Na_2_HPO_4_ ×2H_2_O, 0.02 M glucose). Cells were fixed in the presence of 1% formaldehyde for 20 min at room temperature by addition of 4 ml of formaldehyde solution (50 mM Hepes-KOH pH 7.5, 100 mM NaCl, 1 mM EDTA pH 8.0, 0.5 mM EGTA pH 8.0, 11% formaldehyde). The reaction was stopped by addition of 3 ml of 2 M glycine and incubation for 5 min at room temperature and 15 min on ice. Cells were washed twice in 1× TDB and the cell pellet was snap-frozen in liquid nitrogen. Cells were resuspended in 1 ml of permeabilization buffer (100 mM KCl, 10 mM Tris pH 8.0, 25 mM EDTA) supplemented with protease inhibitors (1.5 mM pepstatin A, 4.25 mM leupeptin, 1.06 mM PMSF, 1.06 mM TLCK) and digitonin (200 μM final concentration) and incubated for 5 min at room temperature. Cells were washed twice in 1× NEBuffer3.1 (NEB, B7003S) and resuspended in 342 μl of 1× NEBuffer3.1. After addition of 38 μl of 1% SDS, and an incubation at 65 °C for 10 min, the SDS was quenched by addition of 43 μl of 10% Triton-X 100 (Sigma) and the incubation was continued at room temperature for 15 min. Another 35 μl of water, 13 μl of 10× NEBuffer3.1 and 100 units of MboI (NEB, R0147M) were added and the chromatin was digested at 37 °C overnight while shaking. To inactivate MboI, the sample was incubated at 65 °C for 20 min. Restriction fragments were biotinylated by supplementing the reaction with 60 μl of fill-in mix (0.25 mM biotin-14-dATP (Life Technologies, 19524016), 0.25 mM dCTP, 0.25 mM dGTP, 0.25 mM dTTP (Fermentas), 40 U of DNA polymerase I, large (Klenow) fragment (NEB, M0210)) and incubation at 23 °C for 4 h. The end-repaired chromatin was transferred to 665 μl of ligation mix (1.8% Triton-X 100, 0.18 mg BSA, 1.8× T4 DNA Ligase Buffer (Invitrogen, 46300018) and 5 μl of T4 DNA ligase (invitrogen, 15224025) were added. The ligation was performed for 4 h at 16 °C with interval shake. Crosslinks were reversed by adding 50 μl of 10 mg/ml proteinase K (65 °C for 4 h) and another addition of 50 μl of 10mg/ml proteinase K, 80 μl of 5M NaCl and 70 μl of 10% SDS (65 °C, overnight).

The DNA was precipitated with ethanol and resuspended in 257 μl of TLE (10 mM Tris-HCl, 0.1 mM EDTA, pH 8.0). SDS was added to a final concentration of 0.1% and the sample was split among two tubes for sonication (Covaris S220; microtubes, 175 W peak incident power, 10% duty factor, 200 cycles per burst, 240 s treatment). The samples were recombined and the volume was adjusted to 300 μl with TLE. Fragments between 100 and 400 bp in size were selected using Agencourt AMPure XP beads (Beckman Coulter), according to the manufacturer’s instructions. The DNA fragments were eluted off the beads in 55 μl of TLE.

For end-repair and biotin removal from unligated ends, 70 μl of end-repair mix was added (1× Ligation buffer (NEB), 357 μM dNTPs, 25U T4 PNK (NEB, M0201), 7.5U T4 DNA polymerase I (NEB, M0203), 2.5U DNA polymerase I, large (Klenow) fragment (NEB, M0210)) and incubated for 30 min at 20 °C and 20 min at 75 °C. To inactivate the enzymes, EDTA was added to a final concentration of 10 mM. To isolate biotin-labelled ligation junctions, 50 μl of 10 mg/ml Dynabeads MyOne Streptavidin C1 (Life Technologies, 65001) were washed with 400 μl of 1× Tween washing buffer (TWB; 5 mM Tris-HCl pH 7.5, 0.5 mM EDTA, 1 M NaCl, 0.05% Tween 20), collected with a magnet, resuspended in 400 μl of 2× binding buffer (10 mM Tris-HCl pH 7.5, 1 mM EDTA, 2 M NaCl) and added to the sample suspended in 330 μl TLE. Biotinylated DNA was bound to the beads by incubating the sample for 15 min at room temperature with slow rotation. Subsequently, the DNA-bound beads were captured with a magnet, washed twice with 400 μl of 1× binding buffer, washed once in 100 μl of 1× TLE T4 ligase buffer and resuspeded in 41 μl of TLE. For polyadenylation, 5 μl of 10× NEBuffer 2.1, 1 μl of 10 mM dATP and 3 μl of 5 U/μl of Klenow fragment (3′→ 5′ exo-) (NEB, M0212) and incubated for 30 min at 37 °C followed by deactivation for 20 min at 65 °C. Beads were reclaimed with a magnet, washed once with 400 μl 1× Quick ligation buffer (NEB, M2200) and resuspended in 46.5 μl of 1× Quick ligation buffer (NEB, M2200). 2.5 μl of DNA Quick ligase (NEB, M2200) and 0.5 μl of 50 μM annealed TruSeq adapters were added and incubated for 1 h at room temperature. Beads were separated on a magnet, resuspended in 400 μl of 1× TWB (5 mM Tris-HCl, 0.5 M EDTA, 1 M NaCl, 0.05% Tween-20) and washed for 5 min at room temperature with rotation. Beads were washed on the magnet with 200 μl 1× binding buffer and 200 μl of 1× NEBuffer 2.1 and resuspended in 20 μl of 1× NEBuffer 2.1. The library was amplified in 8 separate reactions of 50 μl. Per reaction, 1.5 μl of 25 μM TruSeq PCR primer cocktail (TruSeq PCR primer cocktail_F, TruSeq PCR primer cocktail_R; see Supplementary Table 4), 25 μl of 2× Kapa HiFi HotStart Ready Mix (Kapa Biosystems, KR0370) and 21.5 μl of water were added to 2 μl of library bound to the beads. Amplification was performed as follows: 3 min at 95 °C, 5 cycles of 20 s at 98 °C, 30 s at 63 °C and 30 s at 72 °C, 1 cycle of 1 min at 72 °C, hold at 4 °C. The PCR reactions were pooled and the beads were removed from the supernatant using a magnet. The library was purified by addition of 1.5 volumes of Agencourt AMPure XP beads (Beckman Coulter), according to the manufacturer’s instructions. The sample was eluted off beads using 25 μl of 1× TLE buffer, transferred to a fresh tube and the concentration was determined using Qubit (Qubit dsDNA HS Assay Kit, Thermo Fisher) and qPCR (KAPA SYBR FAST qPCR Master Mix, Kapa Biosystems), according to the manufacturer’s instructions. Library size distributions were determined on a 5% polyacrylamide gel. Paired-end 76-bp sequencing was carried out using the Illumina NextSeq 500 system with high and mid output NextSeq 500/550 kits according to the manufacturer’s instructions.

### Mapping of Hi-C reads and generation of interaction matrices

Reads were trimmed at their ligation junction using the truncator of the HiCUP pipeline^37^ (version 0.5.9 devel), mapped using bwa mem (https://arxiv.org/abs/1303.3997) of the bwa kit version 0.7.12-r1039 and those with a quality score *q* > 0 were retained. Reads were mapped to the *T. brucei* Lister 427 genome version 9 (Tb427v9). For each chromosome, this genome contains the core region of two homologous chromosomes only once, whereas both of the the respective heterozygous subtelomeric regions belonging to the two homologous chromosomes are included. During the mapping of Hi-C reads, contigs that displayed alternative variations of an assembled allele were removed (Tb427v9_without_allelic_variants) to keep these loci visible in the Hi-C matrices. The primary analysis of Hi-C reads was performed with HiC-Pro^38^ (version 2.10.0) to visually inspect reproducibility among replicates. Raw matrices were normalized for differences in ploidy (for example, read counts at diploid regions were multiplied by 0.5), balanced by iterative correction using HiC-Pro (default settings) and converted into a homer compatible format^39^ using a custom Python script (see ‘Code availability’). To enable comparisons between different Hi-C experiments, each value in the balanced interaction matrix was divided by the respective column sum.

### Distance-dependent decay of interaction frequencies

To visualize the distance-dependent decay, interaction frequencies between Hi-C bin pairs were grouped on the basis of the linear distance between the pairs, and the distribution of the median interaction frequency across distances was plotted.

### Co-localization of genomic loci

To determine whether a region of interest interacted more or less than expected by chance, the median and mean interaction frequencies were calculated for bins overlapping with the regions of interest. In addition, a ‘background’ interaction frequency was determined by randomly selecting regions of identical size from the same matrix. Significance was determined using Welch’s *t*-test.

To identify changes in DNA–DNA interaction frequencies after deletion of histone variants, the ratio of ‘feature median interaction frequencies’/‘background median interaction frequency’ was determined for 100 randomly selected background regions in wild-type, Δ*H3.V*, Δ*H4.V* and Δ*H3.V*Δ*H4.V* cells. Significance was determined using Welch’s *t*-test. Co-localization analyses were performed using balanced interaction matrices (50-kb resolution). Bins with zero values were excluded from the analyses.

### 4C-like analysis

To visualize interactions between one genomic region (bait) and all other genomic sites, relevant bins were extracted from a 20-kb Hi-C matrix. An average interaction value for every genomic bin was calculated if the bait regions spanned more than one bin.

### Hi-C matrix visualization

Matrices were plotted based on the colour palettes provided by seaborn (https://seaborn.pydata.org). To generate differential heat maps, a pseudo-count of 0.000001 was added to each interaction value of the numerator and denominator matrix before division.

### SMRT sequencing

Genomic DNA was isolated and precipitated from 3 × 10^8^ cells of the *T. brucei* 427 17.13 P10 isolate^40^ using the Blood & Cell Culture DNA Midi Kit (Qiagen), according to the manufacturer’s instructions. In addition, the DNA was purified in a phenol chloroform extraction using Manual Phase Lock Gel 2-ml (heavy/light) tubes (5Prime) and suspended in 100 μl of TE buffer. SMRT library preparation and sequencing was performed at the Icahn School of Medicine at Mount Sinai.

Genomic DNA library preparation and sequencing was performed primarily using the manufacturer’s instructions for the P6-C4 sequencing enzyme and chemistries. In short, ~5 μg gDNA was quantified and diluted to 150 μl using elution buffer (Qiagen) at 33 ng/μl and then sheared to ~20 kb by centrifugation at 4,500 rpm for 50 s using a G-tube spin column (Covaris). The sheared DNA was then re-purified using Agencourt AMPure XP beads (Beckman Coulter) at 0.45×. Next, ~1.6 to 3.2 μg of DNA from each batch was taken into DNA damage and end repair. In brief, the DNA fragments were repaired by adding 21.1 μl of DNA damage repair solution (1× DNA damage repair buffer (1× NAD+, 1 mM ATP and 0.1 mM dNTP) and 1× DNA damage repair mix) and incubation at 37 °C for 20 min. DNA ends were repaired by adding 1× end repair mix to the solution and incubation at 25 °C for 5 min, followed by an additional 0.45× Agencourt AMPure XP purification step. Next, 0.75 μM of blunt adaptor was added to the DNA and 1× template preparation buffer (0.05 mM ATP and 0.75 U/μl T4 DNA ligase) was added to a final volume of 47.5 μl. This solution was incubated at 25 °C overnight, followed by incubation at 65 °C for 10 min to inactivate the ligase. To remove un-ligated DNA fragments, exonuclease cocktail (1.81 U/μl Exo III 18 and 0.18 U/μl Exo VII) was added to the library followed by a 60 min incubation at 37 °C. Two additional 0.45× Agencourt Ampure XP purification steps were performed to remove <2,000-bp molecular weight DNA and organic contaminants.

The size of the library was validated using an Agilent DNA 12000 chip. Before P6-C4-based sequencing, Blue Pippin size selection was applied to remove molecules <7,000 bp. This step was conducted using Sage Science Blue Pippin 0.75% agarose cassettes to select libraries in the range of 7,000–50,000 bp. Primers were annealed to the size-selected SMRTbell at a ratio of 20:1 with the full-length libraries by denaturation (80 °C for 2 min) and slow cooling (0.1 °C/s to 25 °C). The polymerase-template complex was bound to the P6 enzyme using a ratio of 10:1 polymerase to SMRTbell at 0.5 nM for 4 h at 30 °C and then held at 4 °C until ready for magnetic-bead loading. The magnetic-bead loading step was conducted at 4 °C for 60 min. The magnetic-bead-loaded, polymerase-bound SMRTbell libraries were placed onto the RSII machine at a sequencing concentration of 50 pM and configured for a 240-min continuous sequencing run.

### Assembly, post-assembly improvements and genome scaffolding

The 642,583 sequencing reads from wild-type gDNA (seven SMRT cells) were assembled into contigs following the RS_HGAP_Assembly.3 workflow from SMRT Analysis v2.3.0^41^ with default parameters. In brief, a sequence seeding dataset with the longest sequencing reads was pulled apart. The remaining reads were mapped onto them to obtain error-corrected reads through a consensus procedure. The error-corrected reads were assembled by traditional overlap layout consensus with a Celera Assembler^42^. Contig sequences were polished with Quiver and were further joined and extended using PBJelly 2 (PBSuite_15.2.20)^43^. Remaining sequence errors were corrected using iCORN2^44^ with previously published and new gDNA Illumina data available under GSM2586510 (https://www.ncbi.nlm.nih.gov/geo/query/acc.cgi?acc=GSM2586510) and ERS1503958 (http://www.ebi.ac.uk/ena/data/view/ERS1503958), respectively.

For genome scaffolding, all Hi-C reads were combined, mapped onto the error-corrected HGAPv3 contigs and a balanced 10-kb bin heat map matrix of DNA–DNA interactions was generated as previously described. For the scaffolding, only a subset of the matrix that contained contigs larger than 50 kb was considered (Extended Data Fig. 1).

Then, contigs that did not exhibit a gradual distance-dependent decay in DNA–DNA interactions (which suggests that they may have been incorrectly assembled) were broken at the site of the putative mis-assembly. Available pipelines for the scaffolding of contigs based on DNA–DNA interaction frequencies are not designed for genomes that contain large regions of heterozygosity^45,46^; therefore, contigs were manually rearranged and/or inverted so that long-distance DNA interactions—visible as signal away from the diagonal—were reduced. The process of Hi-C read-mapping, heat map generation and contig repositioning or inversion was repeated until no contigs could be identified, the repositioning of which would have further minimized the signal away from the diagonal (Extended Data Fig. 1). To validate our scaffolding approach and to further improve the genome, we ran PBJelly 2, which enabled us to join together several of the contigs that we had placed next to each other and to reduce the number of contigs from 139 to 91. In addition, we compared the obtained Lister 427 scaffold to that of the previously assembled TREU 927 genome and found that the core regions exhibited a high degree of similarity and synteny (Extended Data Fig. 2), which validates our scaffolding approach. The subtelomeric regions are known to be different between the genomes. Finally, we observed that 27 out of the 33 core–subtelomere boundaries in the assembly are spanned by PacBio reads and/or contigs, which supports their linear proximity (see Supplementary Information).

For a comparison of different assembly strategies and an assessment of the genome quality, see Supplementary Information and Supplementary Table 3. The genome-built Tb427v9, which was used for all analyses performed in this study, is available in the European Nucleotide Archive with the ENA study accession number PRJEB18945 (http://www.ebi.ac.uk/ena/data/view/PRJEB18945).

### Genome annotation

Annotations from the *T. brucei* TREU 927 genome were transferred using Companion (https://companion.sanger.ac.uk; accessed 9 January 2017)^47^. In addition, VSG genes were annotated based on similarity (>90% coverage and >95% identity to a VSG gene) of the available VSGnome dataset from the Lister 427 strain^10^ with NCBI-BLAST+ (version 2.2.31+). Overlapping VSG-gene entries were merged and named after the entry with the best bit score. For the identification of putative novel MESs, the assignment of BESs to chromosome ends and the identification of centromeres, see Supplementary Information.

### Chromatin immunoprecipitation with sequencing

Assays were performed as previously described^48^ with minor alterations. For the immunoprecipitation of cell lines with Ty1-tagged proteins (*Ty1-H3.V/*Δ*H3.V*, *Ty1-H4.V* Δ*H4.V/*Δ*H4.V* and *Ty1-SCC1/*Δ*SCC1* cells), 50 μl of Dynabeads protein G (ThermoFisher) was separated on a magnet, the supernatant was removed and the beads were resuspended in 200 μl of PBS-Tween (0.02%) containing 10 μg of BB2 antibody^49^, and then incubated with slow rotation at 4 °C overnight. Antibody-coupled protein G beads were separated on a magnetic rack and washed three times with PBS-Tween (0.02%). Five hundred microlitres of ChIP sample was added, and incubated at 4 °C overnight with slow rotation. For ChIP assessment of the distribution of histone variants, the DNA was fragmented using micrococcal nuclease; for ChIP assessment of the distribution of SCC1, the DNA was sheared using a Covaris S220 instrument before target protein binding. Sequencing reads were mapped using bwa mem and coverage plots were generated using COVERnant (v.0.3.2) (https://github.com/konrad/COVERnant)^13^.

### ATAC-seq

To ensure reproducibility of the assays independent of cell-number variations, all assays were performed with 1 × 10^6^ and 2 × 10^6^ cells. To this end 3 × 10^7^ cells were collected and washed in 30 ml of cold 1× TDB. The pellet was resuspended in 300 μl permeabilization buffer with protease inhibitors, 3 μl of 4 mM digitonin was added and incubated for 5 min at room temperature. The cells were pelleted, resuspended in 600 μl isotonic buffer with protease inhibitors and split in two samples, containing 1 × 10^7^ and 2 × 10^7^ cells, respectively. The transposition reaction was performed by adding 50 μl of transposition mix to the pellet (25 μl TD (2× reaction buffer from Nextera kit), 25 μl TDE1 (Tn5 transposase from Nextera kit), 22.5 μl nuclease-free water) and incubation for 30 min at 37 °C. For the gDNA control, 200 ng of gDNA was treated in the same manner. The DNA samples were purified using Qiagen MinElute PCR Purification Kit and eluted in 10 μl EB (10 mM Tris-HCl, pH 8). The transposed DNA fragments were amplified using the NEBNext High-Fidelity 2× PCR Master Mix (M0541) supplied with 2.5 μl of 25 μM barcoded primers and amplification for 13 cycles. The libraries were purified using AMPure XP beads (Beckman Coulter) according to the manufacturer’s instructions. The library fragment sizes between 150 and 1,000 bp were purified from a 6% polyacrylamide gel. Paired-end 76-bp sequencing was carried out using the Illumina NextSeq 500 system with a high-output NextSeq 500/550 kit, according to the manufacturer’s instructions. Sequencing reads were mapped using bwa mem and coverage plots were generated using COVERnant (v.0.3.2) (https://github.com/konrad/COVERnant)^13^.

### scRNA-seq

*T. brucei* wild-type and Δ*H3.V*Δ*H4.V* cells were sorted (0 cell, 1 cell or 50 cells) using a FACSAria III (BD Biosciences; precision: single-cell; nozzle: 100 μm). A forward-scatter area versus side-scatter area plot was used to gate and sort the cells. *T. brucei* cells were sorted in 48-well plates (Brand) filled with 2.6 μl of 1× lysis buffer (Takara) supplemented with 0.01 μl of RNase inhibitor (40 U/μl; Takara). Immediately after sorting, cells were placed on ice for 5 min and stored at −80 °C.

Lysates from 50 trypanosomes and single trypanosomes were supplemented with 0.2 μl of a 1:2 × 10^6^ (scRNA-seq I) or a 1:20 × 10^6^ (scRNA-seq II) dilution of ERCC Spike-In Control Mix 1 (Thermo Fisher, 4456740). Libraries were prepared using SMART-Seq v.4 Ultra Low Input RNA Kit (Takara) using a quarter of the reagent volumes recommended by the manufacturer. PCR amplification was performed using 26 cycles (scRNA-seq I) or 22 cycles (scRNA-seq II), according to the supplier’s recommendations. cDNA was purified using Agencourt AMPure XP beads (Beckman Coulter) and recovered in 15 μl of elution buffer (Takara). Libraries were quantified using the Qubit 3 Fluorometer with dsDNA Hs Assay kit (Life Technologies) and the quality of the libraries was assessed using a 2100 Bioanalyzer with High Sensitivity DNA kit (Agilent) (Extended Data Fig. 7). Similar to previously published approaches^50^, 0.5 ng of cDNA was subjected to a tagmentation-based protocol (Nextera XT, Illumina) using a quarter of the recommended volumes, 10 min for tagmentation at 55 °C and 1 min extension time during PCR amplification. Libraries were pooled (96 libraries for NextSeq) and sequencing was performed in paired-end mode for 2 × 75 cycles using Illumina’s NextSeq 500. Details of the sequencing results are listed in Supplementary Table 2.

### Analysis of scRNA-seq

The reads were mapped to the combination of the Tb427v9 genome with the ERCC spike-in sequences, using bwa mem, version 0.7.16. The mapped data were processed using samtools^51^ (version 1.8) and MarkDuplicates tool (2.18.3-SNAPSHOT) from Picard (http://broadinstitute.github.io/picard/), and the read counts-to-features were done with bedtools^52^ (version 2.26.0). To assess the quality of the data, for each scRNA-seq experiment the read counts to the following groups were determined: reads mapping to ‘rRNA genes’, reads mapping to ‘protein-coding genes’ (CDS plus 89 bp for the 5′ UTR and 400 bp for the 3′ UTR)^53^, reads mapping to ‘other regions of the genome’ and ‘unmapped reads’. Reads that overlapped ‘rRNA genes’ and ‘protein-coding genes’ features at the same time were excluded from both groups and counted as reads mapping to ‘other regions of the genome’.

To assess the library complexity of the scRNA-seq datasets, the number of genes with ≥ 10 read counts was determined for each cell. The counts per feature group, as well as the number of genes captured in each scRNA-seq experiment, are available in Supplementary Table 2. Only those scRNA-seq datasets with more than 500 genes with ≥ 10 read counts were considered for the quantification of VSG gene expression.

Many VSG genes share a high degree of homology with each other. Therefore, to determine the expression levels for each of the 2,846 VSG genes annotated in Tb427v9, the number of uniquely mapping sequence reads obtained for each VSG gene was normalized to account for differences in uniqueness. The uniqueness of the VSG genes was determined by alignment of an in silico-generated dataset that matched the scRNA-seq datasets in read size and fragment-length distribution. For each cell, the transcript level of an individual VSG gene is shown as a percentage of the total VSG gene transcript level in that cell. Raw and normalized read counts are available in Supplementary Table 2, and a diagram explaining the VSG count normalization procedure is shown in Extended Data Fig. 7.

### Total RNA-seq

Triplicates of *T. brucei* wild-type, Δ*H3.V*, Δ*H4.V* and Δ*H3.V*Δ*H4.V* cells were grown to a density of ~10^6^ cells/ml. Cell concentration was determined nine times for each replicate using a Coulter Cell Counter (Beckman Coulter) and 4.5 × 10^7^ cells were collected from each culture. Cells were washed with 1× TDB, resuspended in 350 μl of buffer RA1 (Macherey-Nagel, NucleoSpin RNA) and 38 μl of 0.1 M DTT and 1 μl of a 1:10 dilution of ERCC Spike-In Control Mix (Thermo Fisher, 4456740) were added. Total RNA was purified from the lysate according to the NucleoSpin RNA kit protocol, and eluted in 30 μl of nuclease-free water. To deplete rRNA, 3.5 μg of total RNA was mixed with 2.6 μl of 5× hybridization buffer (500 mM Tris-HCl pH 7.4, 1 M NaCl), 0.459 pmol of 131 50-bp anti-rRNA oligonucleotides (covering *18S*, *M1*, *M2*, *M3*, *M4*, *M5*, *M6*, *28S* alpha and *28S* beta rRNAs, a kind gift of C. Clayton; for the full list, see Supplementary Table 4) and 2.2 μl of water, denatured at 95 °C for 2 min and slowly (0.1 °C per s) cooled to 37 °C. One microlitre of prewarmed 10× RNaseH digestion buffer (200 mM Tris-HCl, pH 7.4, 500 mM KCl, 40 mM MgCl_2_, 10 mM DTT) and 10 U of RNaseH (ThermoFisher, AM2292) were added and the volume was adjusted to 16 μl with nuclease-free water. The mixture was incubated at 37 °C for 20 min. Residual oligonucleotides were subsequently digested by addition of 2 U of Turbo DNaseI and 5 μl of 10× Turbo DNase reaction buffer (AM2238, Thermo Fisher) in a total reaction volume of 50 μl and incubation at 37 °C for 20 min. The DNase was inactivated by addition of EDTA (15 mM final concentration) and heating at 75 °C for 10 min. The RNA was purified using RNAeasy Minelute columns (Qiagen), according to the manufacturer’s instructions, and eluted in 14 μl of RNase-free water. Double-stranded cDNA was synthesized from 100 ng of rRNA-depleted RNA using the NEBNext Ultra RNA Library Prep Kit for Illumina. The double-stranded cDNA was purified using Agencourt AMPure XP beads (Beckman Coulter) and eluted in 60 or 30 μl of 0.1× TE Buffer, respectively. Libraries were prepared and sequenced as previously described^48^.

### Analysis of RNA-seq data

After adaptor clipping and quality trimming using cutadapt^54^ (version 1.10), the RNA-seq reads were mapped against the *T. brucei* genome using bwa mem of the bwa kit version 0.7.16. Only reads with a quality score *q* > 0 were retained. Feature quantification was performed with bedtools multicov subcommand. Differential gene expression analysis was then conducted with DESeq2 (v.1.20.0)^55^. Features with an adjusted *P* value (calculated based on Wald test and adjusted for multiple testing using the procedure of Benjamini and Hochberg^56^) below 0.1 were considered as differentially expressed. Supplementary Table 1 contains the raw counts for each gene in each individual RNA-seq replicate, as well as the fold change (in log_2_ scale) and *P* value adjusted for each sample versus wild type.

### Fluorescence in situ hybridization

For each FISH assay, 1 × 10^7^ bloodstream-form trypanosomes grown to a density of up to 1 × 10^6^ cells/ml were collected and the cell pellet was washed once with 1× TDB and fixed for 15 min in 1× TDB containing 4% formaldehyde. Cells were washed with 1× TDB once and resuspended in 50 μl of 1× TDB. Gene frames were placed onto microscopy slides to cover an aminopropyltriethoxysilane-coated coverslip. Cells were pipetted onto the framed coverslip and allowed to settle for 5 min by gravity. The sample was washed twice for 2 min with 90 μl of 1× TDB, incubated with quenching solution (1× TDB containing 1 mg/ml NaBH_4_) for 10 min, washed twice with 1× TDB, permeabilized with 70 μl of 1× TDB containing 0.1% NP-40 for 5 min and washed twice with 1× TDB. Next, cells were treated with 1× PBS containing 1 mg/ml RNaseA for 30 min, washed twice with 1× TDB and incubated with 50 μl of a 1:1 dilution of hybridization buffer with 2× SSC for 30 min at room temperature. The labelled probe (see Supplementary Table 4) was diluted to a final concentration of 400 nM in 25 μl of hybridization buffer (50 (v/v) formamide, 10% (w/v) dextran sulfate, 2× SSPE, 250 μg/ml herring sperm DNA). The hybridization buffer was removed from the sample, 25 μl of hybridization solution containing the probe was added and the frame was closed with a plastic lid. The sample was incubated using a thermal block at 90 °C for 5 min and at 37 °C overnight. Next, the samples were washed for 30 min in 30 ml of 50% (v/v) deionized formamide and 2× SSC at 37 °C, for 10 min in 30 ml of 1× SSC at 50 °C, for 10 min in 30 ml of 2× SSC at 50 °C and for 10 min in 30 ml of 4× SSC at room temperature. Subsequently, cells were blocked in P1 buffer (100 mM maleic acid, 150 mM sodium chloride, pH 7.5, 4% BSA, 1% milk) for 1 h, incubated with primary antibody (sheep anti-digoxigenin antigen-binding fragment, Roche, diluted 1:2,000 in P1) for 45–60 min and washed with 0.5% Tween-20 and PBS 4 times for 4 min each. Cells were then incubated with the secondary antibody (Alexa Fluor 488 conjugated donkey anti-sheep IgG (H + L), Life Technologies, A11015, diluted 1:2,000 in P1) for 30–60 min. For further signal amplification, the samples were washed with PBS containing 0.5% Tween-20 4 times for 4 min and incubated with a rabbit anti-donkey IgG (H + L) DyLight 488 (Invitrogen) (diluted 1:2,000 in P1) for 30-60 min. The samples were washed twice for 10 min in 30 ml of PBS containing 0.5% Tween-20 and another 10 min in 30 ml of PBS, each time in a falcon tube on a shaker. Samples were mounted with 36 μl of Vectashield Mounting Medium with DAPI (Biozol) on a microscopy slide and were sealed with nail polish.

### Immunofluorescence

Immunofluorescence was performed as previously described^57^, with minor alterations. Ten million cells per ml (wild type, Δ*H3.V*, Δ*H4.V* and Δ*H3.V*Δ*H4.V*) were suspended in HMI-11 containing 2% formaldehyde, incubated for 5 min at room temperature and washed with 1× TDB. α-Tubulin was stained using the mouse monoclonal antibody Tat1^58^ (1:200) and a secondary Alexa Fluor 594-conjugated chicken anti-mouse IgG (1:350, Invitrogen). VSG-2 was stained using CRD-depleted rabbit anti-VSG-2^40^ (1:1,000) and a secondary Alexa Fluor 488-conjugated donkey anti-rabbit IgG (1:350, Invitrogen).

### Fluorescence microscopy and image analysis

For imaging, a wide-field fluorescence Leica DMI6000 microscope with a mercury metal halide lamp and a HCX PL APO CS 100×/1.47 OIL objective was used. Images were captured with a Leica DFC 360 FX camera. Stacks with 32 slices and 6.3232 μm in height (0.0645 × 0.0644 × 0.1976 μm^3^ voxel size) were captured using identic exposure times for all conditions.

Quantification of ‘large’ telomere clusters was carried out using Imaris 8 software (Oxford Instruments). After segmenting individual nuclei in the DAPI channel, surfaces were rendered for the telomere FISH signal, while setting the quality filter > 1,000. All FISH signals that generated surfaces with a volume > 0.3 μm^3^ were classified as large telomere clusters, and scored.

### FACS flow cytometry

VSG expression on the cell surface was quantified according to a previously published protocol^40^. In brief, 1 × 10^6^ cells were centrifuged in a chilled microcentrifuge at 1,500*g* for 4 min at 4 °C. Cells were resuspended in 100 μl of ice cold HMI-11 and a VSG-specific antibody (anti-VSG-2 or anti-VSG-13)^40^ was added. After 60 min of incubation at 4 °C with gentle shaking, cells were washed three times in 500 μl of ice cold HMI-11, resuspended in 100 μl of cold HMI-11 and incubated with an Alexa Fluor 488-conjugated secondary antibody for 20 min. The cells were washed twice with 500 μl of 1× TDB and finally resuspended in 400 μl of 1× TDB before analysis with a FACSort flow cytometer (Becton Dickinson Biosciences).

To determine the cell-cycle profiles, 5 × 10^6^ cells were collected (10 min, 1,300*g*, 4°C) and washed once with ice-cold 1× TDB. The cells were resuspended in 1 ml ice-cold PBS and 2 mM EDTA and fixed by adding 2.5 ml ice-cold methanol. After a 1-h incubation, cells were washed with 1 ml PBS and EDTA at room temperature and resuspend in 1 ml PBS and EDTA. One microlitre RNaseA (10 μg/μl) and 10 μl propidium iodide (1 μg/μl) were added. The stained cells were analysed with a FACSCalibur (Becton Dickinson) after a 30-min incubation at 37 °C.

### Mapping the site of recombination

A library from Δ*H3.V*Δ*H4.V* gDNA was prepared as described for wild-type gDNA and sequenced using the PacBio Sequel system by diffusion at 5 pM, with v2.1 chemistry and a 10-h movie. Reads > 10 Kb were extracted, split into ~2,500-bp chunks and mapped independently to the genome. Reads that contained chunks that mapped to BES1 and BES15 were kept and mapped again to BES1 and BES15. Based on the observed mapping pattern, a BES1–BES15 hybrid was constructed.

### Code availability

Workflows and custom-made Unix Shell, Python and R scripts have been deposited at Zenodo (10.5281/zenodo.823671). Documentation to reproduce the data analysis is provided.

### Reporting summary

Further information on research design is available in the Nature Research Reporting Summary linked to this paper.

## Online content

Any methods, additional references, Nature Research reporting summaries, source data, statements of data availability and associated accession codes are available at 10.1038/s41586-018-0619-8.

## Supplementary information

Supplementary Information This file contains details on genome assembly.

Reporting Summary

Supplementary Table 1 RNA-seq analysis.

Supplementary Table 2 Single-cell RNA-seq analysis.

Supplementary Table 3 Genome quality assessment.

Supplementary Table 4 Oligo lists.

## Figures and Tables

**Extended Data Table 1:**
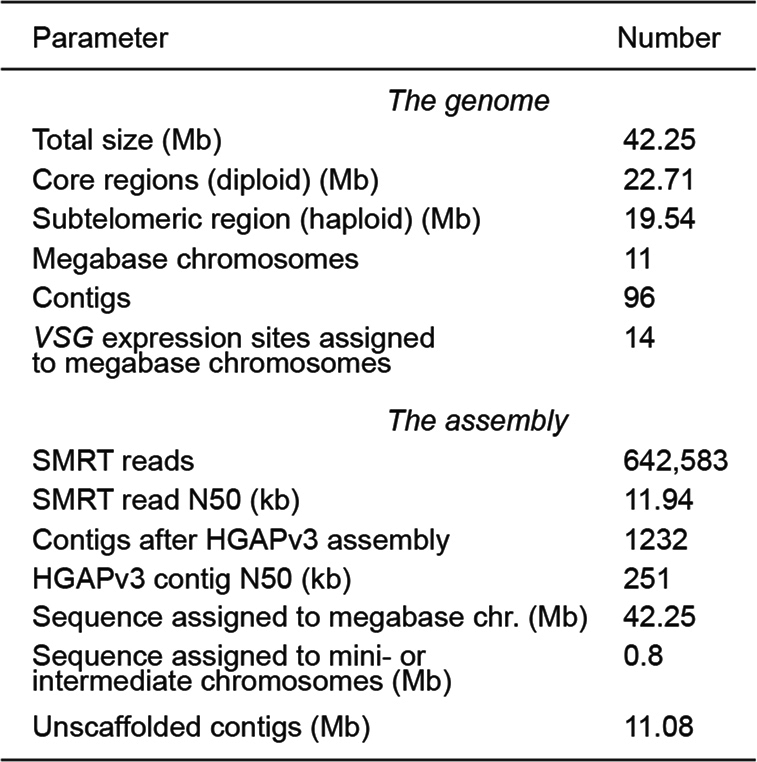
Genome assembly statistics

DNA elements that are smaller than 1 Mb—for example, mini-chromosomes or circular DNA—were not scaffolded for this study, and contribute to the un-scaffolded contigs.

## Data Availability

The RNA-seq, scRNA-seq, ChIP–seq, ATAC-seq and Hi-C sequencing data have been deposited in the Gene Expression Omnibus^59^ and are accessible through GEO Series accession number GSE100896. The raw SMRT sequencing reads and the genome assembly have been deposited in the European Nucleotide Archive and are accessible through ENA study accession number PRJEB18945. All other data are available from the corresponding author upon reasonable request.
