# Supplementary material for: Genome organization and DNA accessibility control antigenic variation in trypanosomes
Source: Nature. 2018 Oct 17;563(7729):121–5. doi: 10.1038/s41586-018-0619-8 (PMC6784898; doi:10.1038/s41586-018-0619-8)
Supplement: Supplementary file 1 — This file contains details on genome assembly. [file 41586_2018_619_MOESM1_ESM.pdf]

nature

SUPPLEMENTARY INFORMATION

https://doi.org/10.1038/s41586-018-0619-8

In the format provided by the authors and unedited.

Genome organization and DNA accessibility control antigenic variation in trypanosomes

Laura S. M. Müller[1,2,3,15], Raúl O. Cosentino[1,2,3,15], Konrad U. Förstner[4,5,6], Julien Guizetti[3,14], Carolin Wedel[3], Noam Kaplan[7], Christian J. Janzen[8], Panagiota Arampatzi[6], Jörg Vogel[9,10], Sascha Steinbiss[11], Thomas D. Otto[11,12], Antoine-Emmanuel Saliba[9], Robert P. Sebra[13] & T. Nicolai Siegel[1,2,3]*

[1]Department of Veterinary Sciences, Experimental Parasitology, Ludwig-Maximilians-Universität München, Munich, Germany. [2]Biomedical Center Munich, Department of Physiological Chemistry, Ludwig-Maximilians-Universität München, Planegg-Martinsried, Germany. [3]Research Center for Infectious Diseases, University of Würzburg, Würzburg, Germany. [4]ZB MED – Information Centre for Life Sciences, Cologne, Germany. [5]TH Köln, Faculty of Information Science and Communication Studies, Cologne, Germany. [6]Core Unit Systems Medicine, Institute of Molecular Infection Biology, University of Würzburg, Würzburg, Germany. [7]Department of Physiology, Biophysics & Systems Biology, Rappaport Faculty of Medicine, Technion Israel Institute of Technology, Haifa, Israel. [8]Department of Cell & Developmental Biology, Biocenter, University of Würzburg, Würzburg, Germany. [9]Helmholtz Institute for RNA-based Infection Research, Würzburg, Germany. [10]RNA Biology Group, Institute of Molecular Infection Biology, University of Würzburg, Würzburg, Germany. [11]Wellcome Trust Sanger Institute, Hinxton, Cambridge, UK. [12]Centre of Immunobiology, Institute of Infection, Immunity & Inflammation, College of Medical, Veterinary and Life Sciences, University of Glasgow, Glasgow, UK. [13]Icahn Institute and Department of Genetics and Genomic Sciences, Icahn School of Medicine at Mount Sinai, New York, NY, USA. [14]Present address: Centre for Infectious Diseases, Parasitology, Heidelberg University Hospital, Heidelberg, Germany. [15]These authors contributed equally: Laura S. M. Müller, Raúl O. Cosentino. *e-mail: n.siegel@lmu.de

© 2018 Springer Nature Limited. All rights reserved.

Comparison of *de novo* assembly strategies. The sequencing reads obtained from seven SMRT cells were assembled using three approaches with default parameters: i) FALCON v0.2.2[1], ii) RS_HGAP_Assembly.3 (HGAPv3)[2] and iii) SPAdes (version3.6.2)[2], combining the SMRT reads with previously published and new gDNA Illumina data available under GSM2230616 (https://www.ncbi.nlm.nih.gov/geo/query/acc.cgi?acc=GSM2230616) and ERS1503958 (http://www.ebi.ac.uk/ena/data/view/ERS1503958), respectively.

SPAdes produced a significantly larger number of contigs with a much smaller N50 than the other two assemblers (52,798 contigs and a N50 of 18 kb, versus 321 and 594 kb, and 1232 and 251 kb in FALCON and HGAPv3, respectively). FALCON produced larger contigs than HGAPv3, but the sum of FALCON assembled contigs (44.4 Mb) was considerably smaller than that obtained with HGAPv3 (65.5 Mb) or SPAdes (63.9 Mb). We suspected that FALCON might have collapsed similar regions, which HGAPv3 did not. To address this question, we determined the number of occurrence of features known to occur several times in the *T. brucei* genome. Specifically, we determined the number of bloodstream form expression site (BES) promoters, procyclin promoters and rRNA promoters in the contigs of the different assemblies and compared them with the number of occurrences in the Tb927 reference genome (version 28) and the draft genome of Lister 427 (Tb427, version 26), both downloaded from the TriTryDB database (tritrypdb.org). On average, the FALCON assembly contained fewer hits than the HGAPv3 assembly and the reference genome, supporting the assumption that in the FALCON assembly many of these regions were collapsed. The HGAPv3 assembly contained more hits than expected compared to the reference genome, which might be related to the presence of allelic variants that HGAPv3 assembled separately.

Finally, using BLAST, we determined the number of *VSGs* present in the different assemblies and compared it to the total Tb427 *VSG*nome composed of 2584 *VSG* genes[3]. Only hits with >90% query coverage and >95% identity were considered positive. The HGAPv3 assembly contained the largest proportion of *VSGs* from the *VSG*nome (96.2%, 2486 different *VSGs*) compared to FALCON (89.5%, 2312) and SPAdes (89.2%, 2306).

In summary, although the total number of contigs of the HGAPv3 assembly was four times larger than that of the FALCON assembly, we proceeded with the HGAPv3 assembly since our analysis suggested that it provided a better resolution of sequence elements occurring at multiple sites across the genome and a better representation of the *VSG*nome.

Scaffolding using DNA-DNA interaction data.

To scaffold the assembled contigs into chromosomes we used DNA-DNA interaction information captured by an genome-wide '*in situ* Hi-C' approach[4], relying on two universal properties of 3D chromosome organization that are directly related with the linear genome: i) within a chromosome, the frequency of interaction between any two DNA regions drops exponentially with their linear distance, ii) Regions located within the same chromosome (although several Mb apart) interact more frequently to each other, than to regions in different chromosomes[5].

Firstly, the mapping of Hi-C reads to individual contigs confirmed a distance-dependent decay in DNA-DNA interactions. Thus, contigs were ordered/scaffolded to minimize long-distance DNA-DNA interactions (visible as signal away from the diagonal in the interaction matrix, Extended Data Fig. 1). The high level of subtelomeric heterozygosity led to the assembly of alleles in separate contigs, which allowed us, with the subsequent Hi-C based-scaffolding, to assemble the *T. brucei* Lister 427 genome with phased subtelomeric regions. Therefore, for the first time most chromosome ends were assembled in an allele-specific manner for a diploid pathogen. The assembly revealed the distribution and location of most *VSG* genes, which are arranged in long haploid-like subtelomeric arrays, sometimes longer than 1Mb (Fig. 1).

Well-defined homozygous-heterozygous boundaries were found for each chromosome, the homozygous cores contained housekeeping genes while the heterozygous subtelomeric regions contained, besides *VSGs* and *pseudo-VSGs*, retrotransposable elements (Fig. 1). The *VSG* repertoire, and the subtelomeric regions in general, differed greatly from the ones present in the TREU 927 genome (Extended Data Fig. 2. By contrast, as expected, the cores were highly similar and syntenic[6], validating our assembly approach.

Identification of putative novel metacyclic expression sites (MES). We used HMMER (version 3.1b2) to generate an hmm profile from published MES promoter sequences[7]. The profile was used to query our *T. brucei* Lister 427 genome assembly using the 'nhmmer' command line tool[8] and yielded the following hits:

Query: *mVSG*-promoter alignment [M=65]
Scores for complete hits:

E-value	score	bias	Sequence	start	end
----------	-------	------	--------------------------	----------	----------
1.4e-18	71.7	1.6	Chr10_5A_Tb427v9	5713	5649
4.2e-18	70.2	1.3	Chr8_5B_Tb427v9	6012	5948
7.3e-16	63.0	6.6	Chr11_5A_Tb427v9	11525	11461
1.3e-14	58.9	4.7	Chr3_5A_Tb427v9	6435	6371
5.6e-13	53.7	2.7	Chr3_5B_Tb427v9	4269	4207
3.9e-06	31.8	3.7	Chr9_5B_Tb427v9	5864	5812
0.00015	26.7	2.1	Chr5_3B_Tb427v9	77151	77095
0.0025	22.8	0.6	Chr10_5B_Tb427v98	11025	10979
0.63	15.1	0.0	Chr9_5A_Tb427v9	5541	5504
1.5	14.0	0.6	Chr8_5B_Tb427v9	307552	307517
5	12.2	2.5	Chr1_3A_Tb427v9	148803	148773
6.3	11.9	0.0	Chr3_core_Tb427v9	239104	239130

The first five hits correspond to the five known *mVSG* promoter nucleotide sequences used to build the profile, which are upstream of *Tb427VSG-397*, *Tb427VSG-1954*, *Tb427VSG-653*, *Tb427VSG-639* and *Tb427VSG-531*. Based on our assembly, these *VSGs* are located at the 5´-end of Chr10_5A, Chr8_5B, Chr11_5A, Chr3_5A, Chr3_5B, respectively. The 6th hit of our list is located upstream (~3.1 kb) of *Tb427VSG-636*, followed by telomeric repeats at the 5´-end of Chr9_5B. The 7th hit is located within Chr5_3B, upstream of *Tb427VSG-582*, but not next to the telomere. This putative MES had been identified previously[3] and the authors suspect that it may not be an active MES, but an ancient MES that recombined with a *VSG* array. The 8th hit is located upstream (~8 kb) of *Tb427VSG-3591* at the end of Chr10_5B. The 9th hit is located upstream (~2.4 kb) of *Tb427VSG-559*, at the end of Chr9_5A. All subsequent hits do not seem to belong to valid MESs based on their locations and surroundings.

In summary, our data suggest that we have identified three new MESs containing the following *VSGs*: *Tb427VSG-636* (Chr9_5B), *Tb427VSG-3591* (Chr10_5B) and *Tb427VSG-559* (Chr9_5A). The *VSGs* of the three new putative MESs (together with the five previously identified) are among the 10 most highly expressed *VSGs* in *T. brucei* Lister 427 metacyclic differentiated cells[7], further supporting our findings.

Assessment of genome assembly quality. To determine the quality of the genome assembly, we used our raw PacBio reads longer than 10 kb to assess, for each base position, whether the assembly at that position is correct, or not assessable. The reads were cut into subreads of 2 kb in length and mapped independently. Mapped subreads were considered as supporting the assembly if the outmost pieces mapped uniquely, in non-repetitive regions, on the same chromosome and strand and within the expected distance (+/- 20% of the expected distance). Due to the high error profile of raw PacBio

reads and the repetitiveness of the genome, chimeras and unreliable merges around repeats may have been generated during the assembly. By mapping 2 kb subreads independently, it is likely they will be placed in the position that is best. If a repeat is spanned by 2 uniquely mapping subreads (with the expected size), the region is regarded as correctly assembled. If a repeat is longer than the reads spanning it, it is regarded as not assessable by unique mapping. Finally, if a region is not spanned by any read (even if mapped repetitively), we regard this region as not assessable by repetitive mapping.

Technically, reads were renamed and cut into subreads with fastaq (https://github.com/sanger-pathogens/Fastaq) and mapped against our *T. brucei* Lister 427 genome assembly using BWA mem[9] (version 0.7.12-r1039, parameter -x pacbio -a -S -P). Subreads from the same PacBio read were considered to be supportive of an assembled region if:

- They map to the same scaffold,
- They map uniquely,
- They map on the same strand,
- Their distance is within the 20% of the expected size,
- They have an alignment score > 1250 (AS:i tag in bam file).

This coverage is represented as a function *f* over each position *x* of the genome. If two subreads satisfy those criteria, and they are at least 4 kb apart, all positions between the two outer most nucleotide coordinates are affected by the associated coverage change. Based on these data, a position is considered as correct if $f(x)\geq 2$. A position is regarded as being not assessable if $f(x)<2$, but when the procedure is repeated allowing repetitive mapping (delivering $f_{rep}(x)$), a position is regarded as being not assessable if $f_{rep}(x)<2$. A custom Perl script was used to analyse the split reads and provide a plot, illustrating the mapping coverage of each genomic position. Results were visually inspected with BAMview[10].

For our analysis the 221,894 raw PacBio reads longer than 10 kb were cut into 1,828,098 subreads ≤ 2 kb. A total of 95% of the subreads mapped to the genomic sequence. For the calculation of coverage statistics, we excluded the unassembled contigs (~11 Mb in size). A total of 40,242,152 bases (of 41,747,005, 96.4%) of the assembly are considered correct with unique mapping. A total of 41,472,183 bases (99,3%) were correctly covered allowing repetitive mapping, leaving only 0.7% of the genome sequence difficult to be assessed. Visual inspection indicated that most of these regions represented assembly gaps, e.g. at centromeric regions. This analysis ensures that if regions are of interest for further analysis, they can be trusted or not.

To assess the accuracy of the homozygous cores and the heterozygous subtelomeres, a similar approach was used. Here, the reads containing subreads spanning the core/subtelomere boundaries were quantified. 27 out of 33 boundaries were supported either by ≥ 10 Pacbio reads or assembled contigs.

BES assignment to chromosome-ends. The HGAPv3 assembly pipeline only placed BES8 onto one of the megabase chromosomes (Chr8_3B). To determine the genomic location of the other BESs, we determined the DNA-DNA interaction frequency between the different BESs and the 100 kb of each chromosome-end. As for the scaffolding of the other contigs, we assumed a distance-depend decay in DNA-DNA interactions. A BES_x was assigned to a chromosome-end_y if the following two conditions were met in each of the twelve iced Hi-C matrices: i) Across all BESs, BES_x is the one with the highest DNA-DNA interaction frequencies to chromosome-end_y, and ii) Reciprocally, chromosome-end_y is the most interacting chromosome-end to BES_x. Based on these criteria the following BESs were assigned: BES12 to the 3´-end of Chr2_core, BES15 to the 3´-end of subtelomere Chr3_3A, BES3 to the 5´-end of the subtelomere Chr4_5B and BES5 to the 5´-end of Chr5_core, and BES1 to the 5’-end of Chr6_core.

Previously, BES1 and BES3 have been mapped to Chr6 and Chr4, respectively[11]. Thus, while our assignments agree with previous results, we were also able to place

three additional BESs and, for the first time, to determine their precise chromosome arm. Visual inspection of 4C-like maps using the different BES as 'bait', like the ones shown in Fig. 3d, confirmed the assignments made. Furthermore, it let us predict the localization for BES7 and BES14, which were not assigned to any chromosome-end during our strict quantification approach.

BES7 and BES14 where previously shown to be on chromosomes bigger than 3.1 Mb[12]. For BES7, we observed strong interactions to the 5´-end of Chr6_core and a gradual decay of interactions to the 3´-end of the chromosome, as expected if it would be located at the 5´-end of Chr6_core. However, given that in our quantification approach the values for BES7 were always smaller than the ones observed for BES1, which is located at the 5´-end of Chr6_core, we choose not to assign BES to any particular chromosome-end. Nonetheless, the 4C-like map suggests that BES7 is located at the homologous 5-subtelomere of Chr6. Regarding BES14, a previous study indicates that it could harbor *Tb427VSG-19* or *Tb427VSG-8*[12]. In the latter case, it would harbor the same *VSG* as BES12. In our genome assembly, the only copy of *Tb427VSG-19* was assembled into a minichromosome-like region (surrounded by 177-bp repeats upstream, and telomeric sequence downstream), which was also found by Cross and collaborators in the Lister 427 single marker cell line[3]. The presence of *Tb427VSG-8* in BES14 could not be confirmed using our assembly because it did not span the 70-bp repeat region downstream of the *pseudo-VSGs* in BES14. However, a 4C-like map using *Tb427VSG-8* as bait suggests that *Tb427VSG-8* is not only located in BES12 but also in BES14. Furthermore, it indicates that BES14 is located downstream of the 3´-end of Chr7_core.

No BESs were assigned to Chr1, Chr9, Chr10 or Chr11, as predicted in a karyotype study performed previously[13]. BES2, BES4, BES10, BES11, BES13 and BES17 are located on intermediate chromosomes[12]. Consequently, we did not observe strong DNA-DNA interactions between those BESs and megabase chromosomes in the 4C-like maps.

Expression sites located on shorter, sub-megabase chromosomes were not scaffolded but included in downstream analyses.

Identification of centromeres. To identify centromeres, we used ChIP-seq data from the kinetochore protein KKT2 generated by Akiyoshi and Gull, 2014 (accession numbers SRX372731 and SRX372732)[14]. Input and ChIP-seq reads were mapped to our Lister 427 genome assembly and a coverage plot of the ratio between them was generated with COVERnant (v0.3.2) (https://github.com/konrad/COVERnant). The ratio plot was visualized using the Integrative Genomics Viewer (IGV)[15] to identify the regions of KKT2 enrichment in each chromosome. In chromosome 1-8, KKT2 enrichment regions agree with the expected location of centromeres based on synteny with the Tb927 strain, for which the centromeric regions are known.

Centromeres for chromosomes 9-11 had been elusive in the genome assembly of the Tb927 strain. As expected, no sites of KKT2 enrichment could be identified in the core regions of chromosomes 9-11 in our *T. brucei* Lister 427 assembly. However, we found regions of KKT2 enrichment in the heterozygous subtelomeric regions. The newly identified sites of KKT2 enrichment contain AT-rich repeats, just like the previously described centromeric regions. They represent regions that could not be bridged in our assembly, indicating that they may be very large, again, just like the previously described centromeric regions, which are between 20 and 120 kb in length[16].

References:

1. Chin, C. S. et al. Phased diploid genome assembly with single-molecule real-time sequencing. *Nat Methods* (2016).
2. Bankevich, A. et al. SPAdes: a new genome assembly algorithm and its applications to single-cell sequencing. *J Comput Biol* **19**, 455-477 (2012).

3. Cross, G. A., Kim, H. S. & Wickstead, B. Capturing the variant surface glycoprotein repertoire (the VSGnome) of Trypanosoma brucei Lister 427. *Mol Biochem Parasitol* **195**, 59-73 (2014).
4. Rao, S. S. et al. A 3D map of the human genome at kilobase resolution reveals principles of chromatin looping. *Cell* **159**, 1665-1680 (2014).
5. Lajoie, B. R., Dekker, J. & Kaplan, N. The Hitchhiker's guide to Hi-C analysis: Practical guidelines. *Methods* **72**, 65-75 (2015).
6. Berriman, M. et al. The genome of the African trypanosome *Trypanosoma brucei. Science* **309**, 416-422 (2005).
7. Kolev, N. G., Ramey-Butler, K., Cross, G. A., Ullu, E. & Tschudi, C. Developmental progression to infectivity in Trypanosoma brucei triggered by an RNA-binding protein. *Science* **338**, 1352-1353 (2012).
8. Wheeler, T. J. & Eddy, S. R. nhmmer: DNA homology search with profile HMMs. *Bioinformatics* **29**, 2487-2489 (2013).
9. Li, H. & Durbin, R. Fast and accurate long-read alignment with Burrows-Wheeler transform. *Bioinformatics* **26**, 589-595 (2010).
10. Carver, T. et al. BamView: visualizing and interpretation of next-generation sequencing read alignments. *Brief Bioinform* **14**, 203-212 (2013).
11. Navarro, M. & Cross, G. A. DNA rearrangements associated with multiple consecutive directed antigenic switches in Trypanosoma brucei. *Mol Cell Biol* **16**, 3615-3625 (1996).
12. Hertz-Fowler, C. et al. Telomeric expression sites are highly conserved in *Trypanosoma brucei. PLoS ONE* **3**, e3527 (2008).
13. Melville, S. E., Leech, V., Navarro, M. & Cross, G. A. The molecular karyotype of the megabase chromosomes of Trypanosoma brucei stock 427. *Mol Biochem Parasitol* **111**, 261-273 (2000).
14. Akiyoshi, B. & Gull, K. Discovery of Unconventional Kinetochores in Kinetoplastids. *Cell* (2014).
15. Robinson, J. T. et al. Integrative genomics viewer. *Nat. Biotechnol.* **29**, 24-26 (2011).
16. Echeverry, M. C., Bot, C., Obado, S. O., Taylor, M. C. & Kelly, J. M. Centromere-associated repeat arrays on Trypanosoma brucei chromosomes are much more extensive than predicted. *BMC Genomics* **13**, 29 (2012).
