# Supplementary material for: Genome organization and DNA accessibility control antigenic variation in trypanosomes
Source: Nature. 2018 Oct 17;563(7729):121–5. doi: 10.1038/s41586-018-0619-8 (PMC6784898; doi:10.1038/s41586-018-0619-8)
Supplement: Supplementary file 2 — Reporting Summary [file 41586_2018_619_MOESM2_ESM.pdf]

nature research

Corresponding author(s): T. Nicolai Siegel

Reporting Summary

Nature Research wishes to improve the reproducibility of the work that we publish. This form provides structure for consistency and transparency in reporting. For further information on Nature Research policies, see Authors & Referees and the Editorial Policy Checklist.

Statistical parameters

When statistical analyses are reported, confirm that the following items are present in the relevant location (e.g. figure legend, table legend, main text, or Methods section).

n/a	Confirmed	
☐	☒	The exact sample size (n) for each experimental group/condition, given as a discrete number and unit of measurement
☐	☒	An indication of whether measurements were taken from distinct samples or whether the same sample was measured repeatedly
☐	☒	The statistical test(s) used AND whether they are one- or two-sided<br>*Only common tests should be described solely by name; describe more complex techniques in the Methods section.*
☒	☐	A description of all covariates tested
☒	☐	A description of any assumptions or corrections, such as tests of normality and adjustment for multiple comparisons
☐	☒	A full description of the statistics including central tendency (e.g. means) or other basic estimates (e.g. regression coefficient) AND variation (e.g. standard deviation) or associated estimates of uncertainty (e.g. confidence intervals)
☐	☒	For null hypothesis testing, the test statistic (e.g. F, t, r) with confidence intervals, effect sizes, degrees of freedom and P value noted<br>*Give P values as exact values whenever suitable.*
☒	☐	For Bayesian analysis, information on the choice of priors and Markov chain Monte Carlo settings
☒	☐	For hierarchical and complex designs, identification of the appropriate level for tests and full reporting of outcomes
☒	☐	Estimates of effect sizes (e.g. Cohen's d, Pearson's r), indicating how they were calculated
☐	☒	Clearly defined error bars<br>*State explicitly what error bars represent (e.g. SD, SE, CI)*

Our web collection on statistics for biologists may be useful.

Software and code

Policy information about availability of computer code

Data collection	no software for data collection was used
Data analysis	Published code<br>Cutadapt (version 1.15); Martin, M. et al. Cutadapt Removes Adapter Sequences From High-Throughput Sequencing Reads. EMBnet.journal 17, 10-12 (2011)<br>bcl2fastq v.20.0.422<br>Fastx package (version 0.0.1.3)<br>HiCUP (version 0.5.9 devel); Wingett, S. et al. HiCUP: pipeline for mapping and processing Hi-C data. F1000Res 4, 1310 (2015)<br>Hi-C pro (version 2.7.10); Servant, N. et al. HiC-Pro: an optimized and flexible pipeline for Hi-C data processing. Genome Biol 16, 259 (2015)<br>COVERnant (version 0.3.2); https://github.com/konrad/COVERnant<br>Samtools (version 1.8)<br>BWA-mem (version 0.7.12-r1039); https://arxiv.org/abs/1303.3997<br>SMRT Analysis (version 2.3.0); Chin, C. S. et al. Nonhybrid, finished microbial genome assemblies from long-read SMRT sequencing data. Nat Methods 10, 563-569 (2013)<br>PBJelly 2 (PBSuite_15.2.20); English, A. C. et al. Mind the gap: upgrading genomes with Pacific Biosciences RS long-read sequencing technology. PLoS One 7, e47768 (2012)<br>iCORN2; Otto, T. D., Sanders, M., Berriman, M. & Newbold, C. Iterative Correction of Reference Nucleotides (iCORN) using second generation sequencing technology. Bioinformatics 26, 1704-1707 (2010)

Companion; Steinbiss, S. et al. Companion: a web server for annotation and analysis of parasite genomes. Nucleic Acids Res 44, W29-34 (2016)
BEDTtools (version 2.26.0); Quinlan, A. R. & Hall, I. M. BEDTools: a flexible suite of utilities for comparing genomic features. Bioinformatics (2010)
DESeq2 (version 1.20.0); Love, M. I., Huber, W. & Anders, S. Moderated estimation of fold change and dispersion for RNA-seq data with DESeq2. Genome Biol 15, 550 (2014)
Seaborn (https://seaborn.pydata.org)
Fiji (https://fiji.sc/)
Imaris (version 8) (http://www.bitplane.com/imaris)

Custom code
The described analysis workflows and required custom made Unix Shell, Python and R scripts were deposited and are accessible at Zenodo (DOI 10.5281/zenodo.823671). All data is available via NCBI GEO (accession GSM2586510) and EBI ENA (accession PRJEB18945).

For manuscripts utilizing custom algorithms or software that are central to the research but not yet described in published literature, software must be made available to editors/reviewers upon request. We strongly encourage code deposition in a community repository (e.g. GitHub). See the Nature Research guidelines for submitting code & software for further information.

Data

Policy information about availability of data

All manuscripts must include a data availability statement. This statement should provide the following information, where applicable:

- Accession codes, unique identifiers, or web links for publicly available datasets
- A list of figures that have associated raw data
- A description of any restrictions on data availability

The RNA-seq, scRNA-seq, ChIP-seq, ATAC-seq and Hi-C sequencing data used in this publication have been deposited in NCBI's Gene Expression Omnibus69 and are accessible through GEO Series accession number GSE100896 (https://www.ncbi.nlm.nih.gov/geo/query/acc.cgi?acc=GSE100896). The raw SMRT sequencing reads and the genome assembly have been deposited in EBI's European Nucleotide Archive and are accessible through ENA Study accession number PRJEB18945 (http://www.ebi.ac.uk/ena/data/view/PRJEB18945). Workflows and custom-made Unix Shell, Python and R scripts have been deposited at Zenodo (https://zenodo.org/record/823671, https://doi.org/10.5281/zenodo.823671). Documentation to reproduce the data analysis is provided.

Field-specific reporting

Please select the best fit for your research. If you are not sure, read the appropriate sections before making your selection.

☒ Life sciences ☐ Behavioural & social sciences ☐ Ecological, evolutionary & environmental sciences

For a reference copy of the document with all sections, see nature.com/authors/policies/ReportingSummary-flat.pdf

Life sciences study design

All studies must disclose on these points even when the disclosure is negative.

Sample size	Sample sizes were not statistically predetermined.
Data exclusions	In the scRNA-seq analysis, only data from cells with more than 500 genes with more than 10 reads per gene were included. Therefore, data from 34 single cells were excluded because they were not matching the criteria.
Replication	All attempts at replication were successful.<br>Hi-C experiments were perfomed in triplicates for each cell line.<br>RNA-seq experiments were performed in triplicates for each isolate.<br>MNase-ChIP-seq experiments were performed in duplicates with an input control for each experiment.<br>Scc1-ChIP experiments were performed in triplicates with an input control for each experiment.<br>ATAC-seq experiments were performed in duplicates with different cell numbers, respectively. Two gDNA samples were included as internal control for accessibility.<br>Single-cell RNA-seq analysis is based on 40 and 408 cells per cell line, respectively.<br>Flow cytometry experiments were performed in triplicates for each cell line.<br>Quantification of telomere clusters (FISH microscopy) was performed in duplicates (total number of cells: 1128).
Randomization	Not relevant for this study as allocation of samples/organisms was not needed or intended.
Blinding	Image acquisition and analysis was done in a blinded fashion.<br>a) For IF images, pictures were taken randomly from the slide without "searching" for a suitable area.<br>b) FISH images were taken and analyzed by an unbiased, external investigator. This person also chose representative images for the publication.<br>For other analyses investigators were not blinded.

Reporting for specific materials, systems and methods

Materials & experimental systems

n/a	Involved in the study	
☒	☐	Unique biological materials
☐	☒	Antibodies
☒	☐	Eukaryotic cell lines
☒	☐	Palaeontology
☒	☐	Animals and other organisms
☒	☐	Human research participants

Methods

n/a	Involved in the study	
☐	☒	ChIP-seq
☐	☒	Flow cytometry
☒	☐	MRI-based neuroimaging

Antibodies

Antibodies used

BB2 antibody
Bastin, P., Bagherzadeh, A., Matthews, K. R. & Gull, K. A novel epitope tag system to study protein targeting and organelle biogenesis in Trypanosoma brucei. Mol. Biochem. Parasitol. 77, 235-239 (1996).

Mouse Tat1 (anti-alpha-tubulin)
Woods, A. et al. Definition of individual components within the cytoskeleton of Trypanosoma brucei by a library of monoclonal antibodies. J Cell Sci 93, 491-500 (1989)

Rabbit anti-VSG-2 (CRD-depleted)
Figueiredo, L. M., Janzen, C. J. & Cross, G. A. M. A histone methyltransferase modulates antigenic variation in African trypanosomes. PLoS Biol. 6, e161 (2008).

Sheep anti-Digoxigenin Fab fragment, Roche, cat: 11214667001, lot: 10392700

Alexa Fluor 488 conjugated donkey anti-Sheep IgG (H+L), life technologies, cat: A11015, lot: 1567206

Rabbit anti-Donkey IgG (H+L) DyLight 488, Invitrogen, cat: SA5-10062, lot: SC2353022

Alexa Flour 594 conjugated chicken anti-mouse IgG, Invitrogen, cat: A21201, lot: 1618343

Validation

Primary antibodies were validated regarding specificity and checked for cross-reactivity as described in the according publications:
BB2 antibody
Bastin, P., Bagherzadeh, A., Matthews, K. R. & Gull, K. A novel epitope tag system to study protein targeting and organelle biogenesis in Trypanosoma brucei. Mol. Biochem. Parasitol. 77, 235-239 (1996).

Mouse Tat1 (anti-alpha-tubulin)
Woods, A. et al. Definition of individual components within the cytoskeleton of Trypanosoma brucei by a library of monoclonal antibodies. J Cell Sci 93, 491-500 (1989)

Rabbit anti-VSG-2 (CRD-depleted)
Figueiredo, L. M., Janzen, C. J. & Cross, G. A. M. A histone methyltransferase modulates antigenic variation in African trypanosomes. PLoS Biol. 6, e161 (2008).

ChIP-seq

Data deposition

☒ Confirm that both raw and final processed data have been deposited in a public database such as GEO.

☒ Confirm that you have deposited or provided access to graph files (e.g. BED files) for the called peaks.

Data access links
May remain private before publication.

GEO Series accession number GSE100896 (https://www.ncbi.nlm.nih.gov/geo/query/acc.cgi?acc=GSE100896)

Files in database submission

H3V ChIP Raw
L1800150_H3_V_Ty_MNase_ChIP_1_R1.fq.gz
L1800150_H3_V_Ty_MNase_ChIP_1_R2.fq.gz
L1800151_H3_V_Ty_MNase_Input_1_R1.fq.gz
L1800151_H3_V_Ty_MNase_Input_1_R2.fq.gz
L1800152_H3_V_Ty_MNase_ChIP_2_R1.fq.gz

L1800152_H3_V_Ty_MNase_ChIP_2_R2.fq.gz
L1800153_H3_V_Ty_MNase_Input_2_R1.fq.gz
L1800153_H3_V_Ty_MNase_Input_2_R2.fq.gz

Output - H3V ChIP
alignment
L1800150_H3_V_Ty_MNase_ChIP_1_to_HGAP3_Tb427v9_bwa.sorted.bam
L1800150_H3_V_Ty_MNase_ChIP_1_to_HGAP3_Tb427v9_bwa.sorted.bam.bai
L1800150_H3_V_Ty_MNase_ChIP_1_to_HGAP3_Tb427v9_bwa.sorted.mapq10.bam
L1800150_H3_V_Ty_MNase_ChIP_1_to_HGAP3_Tb427v9_bwa.sorted.mapq10.bam.bai
L1800151_H3_V_Ty_MNase_Input_1_to_HGAP3_Tb427v9_bwa.sorted.bam
L1800151_H3_V_Ty_MNase_Input_1_to_HGAP3_Tb427v9_bwa.sorted.bam.bai
L1800151_H3_V_Ty_MNase_Input_1_to_HGAP3_Tb427v9_bwa.sorted.mapq10.bam
L1800151_H3_V_Ty_MNase_Input_1_to_HGAP3_Tb427v9_bwa.sorted.mapq10.bam.bai
L1800152_H3_V_Ty_MNase_ChIP_2_to_HGAP3_Tb427v9_bwa.sorted.bam
L1800152_H3_V_Ty_MNase_ChIP_2_to_HGAP3_Tb427v9_bwa.sorted.bam.bai
L1800152_H3_V_Ty_MNase_ChIP_2_to_HGAP3_Tb427v9_bwa.sorted.mapq10.bam
L1800152_H3_V_Ty_MNase_ChIP_2_to_HGAP3_Tb427v9_bwa.sorted.mapq10.bam.bai
L1800153_H3_V_Ty_MNase_Input_2_to_HGAP3_Tb427v9_bwa.sorted.bam
L1800153_H3_V_Ty_MNase_Input_2_to_HGAP3_Tb427v9_bwa.sorted.bam.bai
L1800153_H3_V_Ty_MNase_Input_2_to_HGAP3_Tb427v9_bwa.sorted.mapq10.bam
L1800153_H3_V_Ty_MNase_Input_2_to_HGAP3_Tb427v9_bwa.sorted.mapq10.bam.bai

H3V ChIP Wiggle files (coverage plots)
ws1ss1_denominator_L1800151_H3_V_Ty_MNase_Input_1_to_HGAP3_Tb427v9_bwa.sorted.mapq10.wig
ws1ss1_denominator_L1800153_H3_V_Ty_MNase_Input_2_to_HGAP3_Tb427v9_bwa.sorted.mapq10.wig
ws1ss1_numerator_L1800150_H3_V_Ty_MNase_ChIP_1_to_HGAP3_Tb427v9_bwa.sorted.mapq10.wig
ws1ss1_numerator_L1800152_H3_V_Ty_MNase_ChIP_2_to_HGAP3_Tb427v9_bwa.sorted.mapq10.wig
ws1ss1_ratio_L1800151_H3_V_Ty_MNase_Input_1_to_HGAP3_Tb427v9_bwa.sorted.mapq10_vs_L1800150_H3_V_Ty_MNase_ChIP_1_to_HGAP3_Tb427v9_bwa.sorted.mapq10.wig
ws1ss1_ratio_L1800153_H3_V_Ty_MNase_Input_2_to_HGAP3_Tb427v9_bwa.sorted.mapq10_vs_L1800152_H3_V_Ty_MNase_ChIP_2_to_HGAP3_Tb427v9_bwa.sorted.mapq10.wig
ws2001ss501_denominator_L1800151_H3_V_Ty_MNase_Input_1_to_HGAP3_Tb427v9_bwa.sorted.mapq10.wig
ws2001ss501_denominator_L1800153_H3_V_Ty_MNase_Input_2_to_HGAP3_Tb427v9_bwa.sorted.mapq10.wig
ws2001ss501_numerator_L1800150_H3_V_Ty_MNase_ChIP_1_to_HGAP3_Tb427v9_bwa.sorted.mapq10.wi
ws2001ss501_numerator_L1800152_H3_V_Ty_MNase_ChIP_2_to_HGAP3_Tb427v9_bwa.sorted.mapq10.wi
ws2001ss501_ratio_L1800151_H3_V_Ty_MNase_Input_1_to_HGAP3_Tb427v9_bwa.sorted.mapq10_vs_L1800150_H3_V_Ty_MNase_ChIP_1_to_HGAP3_Tb427v9_bwa.sorted.mapq10.wig
ws2001ss501_ratio_L1800153_H3_V_Ty_MNase_Input_2_to_HGAP3_Tb427v9_bwa.sorted.mapq10_vs_L1800152_H3_V_Ty_MNase_ChIP_2_to_HGAP3_Tb427v9_bwa.sorted.mapq10.wig
ws501ss101_denominator_L1800151_H3_V_Ty_MNase_Input_1_to_HGAP3_Tb427v9_bwa.sorted.mapq10.wig
ws501ss101_denominator_L1800153_H3_V_Ty_MNase_Input_2_to_HGAP3_Tb427v9_bwa.sorted.mapq10.wig
ws501ss101_numerator_L1800150_H3_V_Ty_MNase_ChIP_1_to_HGAP3_Tb427v9_bwa.sorted.mapq10.wig
ws501ss101_numerator_L1800152_H3_V_Ty_MNase_ChIP_2_to_HGAP3_Tb427v9_bwa.sorted.mapq10.wig
ws501ss101_ratio_L1800151_H3_V_Ty_MNase_Input_1_to_HGAP3_Tb427v9_bwa.sorted.mapq10_vs_L1800150_H3_V_Ty_MNase_ChIP_1_to_HGAP3_Tb427v9_bwa.sorted.mapq10.wig
ws501ss101_ratio_L1800153_H3_V_Ty_MNase_Input_2_to_HGAP3_Tb427v9_bwa.sorted.mapq10_vs_L1800152_H3_V_Ty_MNase_ChIP_2_to_HGAP3_Tb427v9_bwa.sorted.mapq10.wig

H4V ChIP Raw
L1800154_H4_V_Ty_MNase_ChIP_1_R1.fq.gz
L1800154_H4_V_Ty_MNase_ChIP_1_R2.fq.gz
L1800155_H4_V_Ty_MNase_Input_1_R1.fq.gz
L1800155_H4_V_Ty_MNase_Input_1_R2.fq.gz
L1800156_H4_V_Ty_MNase_ChIP_2_R1.fq.gz
L1800156_H4_V_Ty_MNase_ChIP_2_R2.fq.gz
L1800157_H4_V_Ty_MNase_Input_2_R1.fq.gz
L1800157_H4_V_Ty_MNase_Input_2_R2.fq.gz

Output H4V ChIP
Alignment
L1800154_H4_V_Ty_MNase_ChIP_1_to_HGAP3_Tb427v9_bwa.sorted.bam
L1800154_H4_V_Ty_MNase_ChIP_1_to_HGAP3_Tb427v9_bwa.sorted.bam.bai
L1800154_H4_V_Ty_MNase_ChIP_1_to_HGAP3_Tb427v9_bwa.sorted.mapq10.bam
L1800154_H4_V_Ty_MNase_ChIP_1_to_HGAP3_Tb427v9_bwa.sorted.mapq10.bam.bai
L1800155_H4_V_Ty_MNase_Input_1_to_HGAP3_Tb427v9_bwa.sorted.bam
L1800155_H4_V_Ty_MNase_Input_1_to_HGAP3_Tb427v9_bwa.sorted.bam.bai
L1800155_H4_V_Ty_MNase_Input_1_to_HGAP3_Tb427v9_bwa.sorted.mapq10.bam
L1800155_H4_V_Ty_MNase_Input_1_to_HGAP3_Tb427v9_bwa.sorted.mapq10.bam.bai
L1800156_H4_V_Ty_MNase_ChIP_2_to_HGAP3_Tb427v9_bwa.sorted.bam
L1800156_H4_V_Ty_MNase_ChIP_2_to_HGAP3_Tb427v9_bwa.sorted.bam.bai
L1800156_H4_V_Ty_MNase_ChIP_2_to_HGAP3_Tb427v9_bwa.sorted.mapq10.bam
L1800156_H4_V_Ty_MNase_ChIP_2_to_HGAP3_Tb427v9_bwa.sorted.mapq10.bam.bai
L1800157_H4_V_Ty_MNase_Input_2_to_HGAP3_Tb427v9_bwa.sorted.bam
L1800157_H4_V_Ty_MNase_Input_2_to_HGAP3_Tb427v9_bwa.sorted.bam.bai

L1800157_H4_V_Ty_MNase_Input_2_to_HGAP3_Tb427v9_bwa.sorted.mapq10.bam
L1800157_H4_V_Ty_MNase_Input_2_to_HGAP3_Tb427v9_bwa.sorted.mapq10.bam.bai

H4V ChIPs Wiggle files (coverage plots)
ws1ss1_denominator_L1800155_H4_V_Ty_MNase_Input_1_to_HGAP3_Tb427v9_bwa.sorted.mapq10.wig
ws1ss1_denominator_L1800157_H4_V_Ty_MNase_Input_2_to_HGAP3_Tb427v9_bwa.sorted.mapq10.wig
ws1ss1_numerator_L1800154_H4_V_Ty_MNase_ChIP_1_to_HGAP3_Tb427v9_bwa.sorted.mapq10.wig
ws1ss1_numerator_L1800156_H4_V_Ty_MNase_ChIP_2_to_HGAP3_Tb427v9_bwa.sorted.mapq10.wig
ws1ss1_ratio_L1800155_H4_V_Ty_MNase_Input_1_to_HGAP3_Tb427v9_bwa.sorted.mapq10_vs_L1800154_H4_V_Ty_MNase_ChIP_1_to_HGAP3_Tb427v9_bwa.sorted.mapq10.wig
ws1ss1_ratio_L1800157_H4_V_Ty_MNase_Input_2_to_HGAP3_Tb427v9_bwa.sorted.mapq10_vs_L1800156_H4_V_Ty_MNase_ChIP_2_to_HGAP3_Tb427v9_bwa.sorted.mapq10.wig
ws2001ss501_denominator_L1800155_H4_V_Ty_MNase_Input_1_to_HGAP3_Tb427v9_bwa.sorted.mapq10.wig
ws2001ss501_denominator_L1800157_H4_V_Ty_MNase_Input_2_to_HGAP3_Tb427v9_bwa.sorted.mapq10.wig
ws2001ss501_numerator_L1800154_H4_V_Ty_MNase_ChIP_1_to_HGAP3_Tb427v9_bwa.sorted.mapq10.wig
ws2001ss501_numerator_L1800156_H4_V_Ty_MNase_ChIP_2_to_HGAP3_Tb427v9_bwa.sorted.mapq10.wig
ws2001ss501_ratio_L1800155_H4_V_Ty_MNase_Input_1_to_HGAP3_Tb427v9_bwa.sorted.mapq10_vs_L1800154_H4_V_Ty_MNase_ChIP_1_to_HGAP3_Tb427v9_bwa.sorted.mapq10.wig
ws2001ss501_ratio_L1800157_H4_V_Ty_MNase_Input_2_to_HGAP3_Tb427v9_bwa.sorted.mapq10_vs_L1800156_H4_V_Ty_MNase_ChIP_2_to_HGAP3_Tb427v9_bwa.sorted.mapq10.wig
ws501ss101_denominator_L1800155_H4_V_Ty_MNase_Input_1_to_HGAP3_Tb427v9_bwa.sorted.mapq10.wig
ws501ss101_denominator_L1800157_H4_V_Ty_MNase_Input_2_to_HGAP3_Tb427v9_bwa.sorted.mapq10.wig
ws501ss101_numerator_L1800154_H4_V_Ty_MNase_ChIP_1_to_HGAP3_Tb427v9_bwa.sorted.mapq10.wig
ws501ss101_numerator_L1800156_H4_V_Ty_MNase_ChIP_2_to_HGAP3_Tb427v9_bwa.sorted.mapq10.wig
ws501ss101_ratio_L1800155_H4_V_Ty_MNase_Input_1_to_HGAP3_Tb427v9_bwa.sorted.mapq10_vs_L1800154_H4_V_Ty_MNase_ChIP_1_to_HGAP3_Tb427v9_bwa.sorted.mapq10.wig
ws501ss101_ratio_L1800157_H4_V_Ty_MNase_Input_2_to_HGAP3_Tb427v9_bwa.sorted.mapq10_vs_L1800156_H4_V_Ty_MNase_ChIP_2_to_HGAP3_Tb427v9_bwa.sorted.mapq10.wig

Scc1 ChIP Raw
L1800343_Scc1_Ty1_wt_soni_ChIP_0_R1.fq.gz
L1800343_Scc1_Ty1_wt_soni_ChIP_0_R2.fq.gz
L1800344_Scc1_Ty1_wt_soni_Input_0_R1.fq.gz
L1800344_Scc1_Ty1_wt_soni_Input_0_R2.fq.gz
L1800468_Scc1_Ty1_wt_soni_ChIP_1_R1.fq.gz
L1800468_Scc1_Ty1_wt_soni_ChIP_1_R2.fq.gz
L1800469_Scc1_Ty1_wt_soni_Input_1_R1.fq.gz
L1800469_Scc1_Ty1_wt_soni_Input_1_R2.fq.gz
L1800474_Scc1_Ty1_wt_soni_ChIP_2_R1.fq.gz
L1800474_Scc1_Ty1_wt_soni_ChIP_2_R2.fq.gz
L1800475_Scc1_Ty1_wt_soni_Input_2_R1.fq.gz
L1800475_Scc1_Ty1_wt_soni_Input_2_R2.fq.gz

Output Scc1 ChIP
Alignment
L1800343_Scc1_Ty1_wt_soni_ChIP_0_to_HGAP3_Tb427v9_bwa.sorted.bam
L1800343_Scc1_Ty1_wt_soni_ChIP_0_to_HGAP3_Tb427v9_bwa.sorted.bam.bai
L1800343_Scc1_Ty1_wt_soni_ChIP_0_to_HGAP3_Tb427v9_bwa.sorted.mapq10.bam
L1800343_Scc1_Ty1_wt_soni_ChIP_0_to_HGAP3_Tb427v9_bwa.sorted.mapq10.bam.bai
L1800344_Scc1_Ty1_wt_soni_Input_0_to_HGAP3_Tb427v9_bwa.sorted.bam
L1800344_Scc1_Ty1_wt_soni_Input_0_to_HGAP3_Tb427v9_bwa.sorted.bam.bai
L1800344_Scc1_Ty1_wt_soni_Input_0_to_HGAP3_Tb427v9_bwa.sorted.mapq10.bam
L1800344_Scc1_Ty1_wt_soni_Input_0_to_HGAP3_Tb427v9_bwa.sorted.mapq10.bam.bai
L1800468_Scc1_Ty1_wt_soni_ChIP_1_to_HGAP3_Tb427v9_bwa.sorted.bam
L1800468_Scc1_Ty1_wt_soni_ChIP_1_to_HGAP3_Tb427v9_bwa.sorted.bam.bai
L1800468_Scc1_Ty1_wt_soni_ChIP_1_to_HGAP3_Tb427v9_bwa.sorted.mapq10.bam
L1800468_Scc1_Ty1_wt_soni_ChIP_1_to_HGAP3_Tb427v9_bwa.sorted.mapq10.bam.bai
L1800469_Scc1_Ty1_wt_soni_Input_1_to_HGAP3_Tb427v9_bwa.sorted.bam
L1800469_Scc1_Ty1_wt_soni_Input_1_to_HGAP3_Tb427v9_bwa.sorted.bam.bai
L1800469_Scc1_Ty1_wt_soni_Input_1_to_HGAP3_Tb427v9_bwa.sorted.mapq10.bam
L1800469_Scc1_Ty1_wt_soni_Input_1_to_HGAP3_Tb427v9_bwa.sorted.mapq10.bam.bai
L1800474_Scc1_Ty1_wt_soni_ChIP_2_to_HGAP3_Tb427v9_bwa.sorted.bam
L1800474_Scc1_Ty1_wt_soni_ChIP_2_to_HGAP3_Tb427v9_bwa.sorted.bam.bai
L1800474_Scc1_Ty1_wt_soni_ChIP_2_to_HGAP3_Tb427v9_bwa.sorted.mapq10.bam
L1800474_Scc1_Ty1_wt_soni_ChIP_2_to_HGAP3_Tb427v9_bwa.sorted.mapq10.bam.bai
L1800475_Scc1_Ty1_wt_soni_Input_2_to_HGAP3_Tb427v9_bwa.sorted.bam
L1800475_Scc1_Ty1_wt_soni_Input_2_to_HGAP3_Tb427v9_bwa.sorted.bam.bai
L1800475_Scc1_Ty1_wt_soni_Input_2_to_HGAP3_Tb427v9_bwa.sorted.mapq10.bam
L1800475_Scc1_Ty1_wt_soni_Input_2_to_HGAP3_Tb427v9_bwa.sorted.mapq10.bam.bai

Wiggle files Scc1 ChIP (coverage plots)
ws1ss1_denominator_L1800344_Scc1_Ty1_wt_soni_Input_0_to_HGAP3_Tb427v9_bwa.sorted.mapq10.wig
ws1ss1_denominator_L1800469_Scc1_Ty1_wt_soni_Input_1_to_HGAP3_Tb427v9_bwa.sorted.mapq10.wig
ws1ss1_denominator_L1800475_Scc1_Ty1_wt_soni_Input_2_to_HGAP3_Tb427v9_bwa.sorted.mapq10.wig
ws1ss1_numerator_L1800343_Scc1_Ty1_wt_soni_ChIP_0_to_HGAP3_Tb427v9_bwa.sorted.mapq10.wig

ws1ss1_numerator_L1800468_Scc1_Ty1_wt_soni_ChIP_1_to_HGAP3_Tb427v9_bwa.sorted.mapq10.wig
ws1ss1_numerator_L1800474_Scc1_Ty1_wt_soni_ChIP_2_to_HGAP3_Tb427v9_bwa.sorted.mapq10.wig
ws1ss1_ratio_L1800344_Scc1_Ty1_wt_soni_Input_0_to_HGAP3_Tb427v9_bwa.sorted.mapq10_vs_L1800343_Scc1_Ty1_wt_soni_ChIP_0_to_HGAP3_Tb427v9_bwa.sorted.mapq10.wig
ws1ss1_ratio_L1800469_Scc1_Ty1_wt_soni_Input_1_to_HGAP3_Tb427v9_bwa.sorted.mapq10_vs_L1800468_Scc1_Ty1_wt_soni_ChIP_1_to_HGAP3_Tb427v9_bwa.sorted.mapq10.wig
ws1ss1_ratio_L1800475_Scc1_Ty1_wt_soni_Input_2_to_HGAP3_Tb427v9_bwa.sorted.mapq10_vs_L1800474_Scc1_Ty1_wt_soni_ChIP_2_to_HGAP3_Tb427v9_bwa.sorted.mapq10.wig
ws2001ss501_denominator_L1800344_Scc1_Ty1_wt_soni_Input_0_to_HGAP3_Tb427v9_bwa.sorted.mapq10.wig
ws2001ss501_denominator_L1800469_Scc1_Ty1_wt_soni_Input_1_to_HGAP3_Tb427v9_bwa.sorted.mapq10.wig
ws2001ss501_denominator_L1800475_Scc1_Ty1_wt_soni_Input_2_to_HGAP3_Tb427v9_bwa.sorted.mapq10.wig
ws2001ss501_numerator_L1800343_Scc1_Ty1_wt_soni_ChIP_0_to_HGAP3_Tb427v9_bwa.sorted.mapq10.wig
ws2001ss501_numerator_L1800468_Scc1_Ty1_wt_soni_ChIP_1_to_HGAP3_Tb427v9_bwa.sorted.mapq10.wig
ws2001ss501_numerator_L1800474_Scc1_Ty1_wt_soni_ChIP_2_to_HGAP3_Tb427v9_bwa.sorted.mapq10.wig
ws2001ss501_ratio_L1800344_Scc1_Ty1_wt_soni_Input_0_to_HGAP3_Tb427v9_bwa.sorted.mapq10_vs_L1800343_Scc1_Ty1_wt_soni_ChIP_0_to_HGAP3_Tb427v9_bwa.sorted.mapq10.wig
ws2001ss501_ratio_L1800469_Scc1_Ty1_wt_soni_Input_1_to_HGAP3_Tb427v9_bwa.sorted.mapq10_vs_L1800468_Scc1_Ty1_wt_soni_ChIP_1_to_HGAP3_Tb427v9_bwa.sorted.mapq10.wig
ws2001ss501_ratio_L1800475_Scc1_Ty1_wt_soni_Input_2_to_HGAP3_Tb427v9_bwa.sorted.mapq10_vs_L1800474_Scc1_Ty1_wt_soni_ChIP_2_to_HGAP3_Tb427v9_bwa.sorted.mapq10.wig
ws501ss101_denominator_L1800344_Scc1_Ty1_wt_soni_Input_0_to_HGAP3_Tb427v9_bwa.sorted.mapq10.wig
ws501ss101_denominator_L1800469_Scc1_Ty1_wt_soni_Input_1_to_HGAP3_Tb427v9_bwa.sorted.mapq10.wig
ws501ss101_denominator_L1800475_Scc1_Ty1_wt_soni_Input_2_to_HGAP3_Tb427v9_bwa.sorted.mapq10.wig
ws501ss101_numerator_L1800343_Scc1_Ty1_wt_soni_ChIP_0_to_HGAP3_Tb427v9_bwa.sorted.mapq10.wig
ws501ss101_numerator_L1800468_Scc1_Ty1_wt_soni_ChIP_1_to_HGAP3_Tb427v9_bwa.sorted.mapq10.wig
ws501ss101_numerator_L1800474_Scc1_Ty1_wt_soni_ChIP_2_to_HGAP3_Tb427v9_bwa.sorted.mapq10.wig
ws501ss101_ratio_L1800344_Scc1_Ty1_wt_soni_Input_0_to_HGAP3_Tb427v9_bwa.sorted.mapq10_vs_L1800343_Scc1_Ty1_wt_soni_ChIP_0_to_HGAP3_Tb427v9_bwa.sorted.mapq10.wig
ws501ss101_ratio_L1800469_Scc1_Ty1_wt_soni_Input_1_to_HGAP3_Tb427v9_bwa.sorted.mapq10_vs_L1800468_Scc1_Ty1_wt_soni_ChIP_1_to_HGAP3_Tb427v9_bwa.sorted.mapq10.wig
ws501ss101_ratio_L1800475_Scc1_Ty1_wt_soni_Input_2_to_HGAP3_Tb427v9_bwa.sorted.mapq10_vs_L1800474_Scc1_Ty1_wt_soni_ChIP_2_to_HGAP3_Tb427v9_bwa.sorted.mapq10.wig

ChIP Scripts
ChIP_Scc1_Ty_v9_mapping_covernant.sh
MNase_ChIP_H3V_Ty_v9_mapping_covernant.sh
MNase_ChIP_H4V_Ty_v9_mapping_covernant.sh
extract.sh
ratio.sh
run_alignment.sh

csv files (for generation of metaplots)
2018-04-19_cTTS_2.csv
2018-04-19_sTSS_2.csv

Genome browser session
(e.g. UCSC)

not used

Methodology

Replicates

MNase-ChIP-seq experiments were performed in duplicates with an input control for each experiment.
Scc1-ChIP experiments were performed in triplicates with an input control for each experiment.

Sequencing depth

L1800150_H3_V_Ty_MNase_ChIP_1: NextSeq 500 Mid Output Kit, 150 cycles; total number of paired-end reads: 2840144; read length: 76 bp; paired-end
L1800151_H3_V_Ty_MNase_Input_1: NextSeq 500 Mid Output Kit, 150 cycles; total number of paired-end reads: 4176616; read length: 76 bp; paired-end
L1800152_H3_V_Ty_MNase_ChIP_2: NextSeq 500 Mid Output Kit, 150 cycles; total number of paired-end reads: 3116324; read length: 76 bp; paired-end
L1800153_H3_V_Ty_MNase_Input_2: NextSeq 500 Mid Output Kit, 150 cycles; total number of paired-end reads: 3979824; read length: 76 bp; paired-end
L1800154_H4_V_Ty_MNase_ChIP_1: NextSeq 500 Mid Output Kit, 150 cycles; total number of paired-end reads: 3582319; read length: 76 bp; paired-end
L1800155_H4_V_Ty_MNase_Input_1: NextSeq 500 Mid Output Kit, 150 cycles; total number of paired-end reads: 4636597; read length: 76 bp; paired-end
L1800156_H4_V_Ty_MNase_ChIP_2_R1: NextSeq 500 Mid Output Kit, 150 cycles; total number of paired-end reads: 3924511; read length: 76 bp; paired-end
L1800157_H4_V_Ty_MNase_Input_2_R1: NextSeq 500 Mid Output Kit, 150 cycles; total number of paired-end reads: 4692475; read length: 76 bp; paired-end
L1800343_Scc1_Ty1_wt_soni_ChIP_0: NextSeq 500 Mid Output Kit, 150 cycles; total number of paired-end reads: 9264521; read length: 76 bp; paired-end
L1800344_Scc1_Ty1_wt_soni_Input_0: NextSeq 500 Mid Output Kit, 150 cycles; total number of paired-end reads: 13017782; read length: 76 bp; paired-end
L1800468_Scc1_Ty1_wt_soni_ChIP_1: NextSeq 500 Mid Output Kit, 150 cycles; total number of paired-end reads: 6538932; read length: 76 bp; paired-end

	L1800469_Scc1_Ty1_wt_soni_Input_1: NextSeq 500 Mid Output Kit, 150 cycles; total number of paired-end reads: 6910598; read length: 76 bp; paired-end<br>L1800474_Scc1_Ty1_wt_soni_ChIP_2: NextSeq 500 Mid Output Kit, 150 cycles; total number of paired-end reads: 6370430; read length: 76 bp; paired-end<br>L1800475_Scc1_Ty1_wt_soni_Input_2: NextSeq 500 Mid Output Kit, 150 cycles; total number of paired-end reads: 5353926; read length: 76 bp; paired-end
Antibodies	All ChIP-seq targets were Ty1-taggged and therefore pulled down with a BB2 Antibody (A novel epitope tag system to study protein targeting and organelle biogenesis in Trypanosoma brucei.<br>Bastin P, Bagherzadeh Z, Matthews KR, Gull K<br>Mol Biochem Parasitol. 1996 May; 77(2):235-9.)
Peak calling parameters	For all datasets, sequencing reads were mapped using bwa mem (https://arxiv.org/abs/1303.3997), version 0.7.12-r1039, converted to bam using samtools-1.8 view –b, sorted using samtools-1.8 sort and indexed using samtools index. If required, uniquely mapped reads were extracted using samtools view –bq 10. Coverage plots were generated using COVERnant (https://github.com/konrad/COVERnant).
Data quality	Quality trimming of reads was performed using the Fastx package (version 0.0.1.3). FDR5% were not determined as no peak calling was perfomed in this study.
Software	Cutadapt (version 1.15); Martin, M. et al. Cutadapt Removes Adapter Sequences From High-Throughput Sequencing Reads. EMBnet.journal 17, 10-12 (2011)<br>Fastx package (version 0.0.1.3)<br>COVERnant (version 0.3.2); https://github.com/konrad/COVERnant<br>Samtools (version 1.8)<br>BWA-mem (version 0.7.12-r1039); https://arxiv.org/abs/1303.3997<br>custom shell scripts combining the above tools are available at: Zenodo (DOI 10.5281/zenodo.823671)

Flow Cytometry

Plots

Confirm that:

☒ The axis labels state the marker and fluorochrome used (e.g. CD4-FITC).

☒ The axis scales are clearly visible. Include numbers along axes only for bottom left plot of group (a 'group' is an analysis of identical markers).

☒ All plots are contour plots with outliers or pseudocolor plots.

☒ A numerical value for number of cells or percentage (with statistics) is provided.

Methodology

Sample preparation	1x10*6 cells were centrifuged in a chilled microtube at 1,500 g for 4 min at 4 °C. Cells were resuspended in 100 ?l of ice cold HMI-11 and a VSG-specific antibody (Anti-VSG-2 or Anti-VSG-13; Figueiredo et al. (2008) was added. After 60 min of incubation at 4 °C with gentle shaking, cells were washed three times in 500 ?l of ice cold HMI-11, resuspended in 100 ?l of cold HMI-11 and incubated with an Alexa Fluor 488-conjugated secondary antibody for 20 min. The cells were washed twice with 500 ?l of TDB.
Instrument	BD FACSCalibur type C4A, Becton Dickinson
Software	CellQuest Pro, Version 6.0
Cell population abundance	Post-sort fractions were not used in this study
Gating strategy	Trypanosomes are very homogenous. Only one population was gated in the SSC/FSC window. None-stained WT cells define the negative control. Stained WT cells define the positive population.

☒ Tick this box to confirm that a figure exemplifying the gating strategy is provided in the Supplementary Information.
